# Gender gaps in patience, risk-taking, trust, and prosociality are smaller among younger cohorts

**DOI:** 10.1093/pnasnexus/pgag255

**Published:** 2026-08-11

**Authors:** Rainer Kotschy, Uwe Sunde

**Affiliations:** National Bureau of Economic Research, 1050 Massachusetts Avenue, Cambridge, MA 02138, USA; Department of Global Health and Population, Harvard T.H. Chan School of Public Health, Boston, 677 Huntington Avenue, Boston, MA 02115, USA; CESifo, Munich, Poschingerstr. 5, Munich 81679, Bavaria, Germany; CESifo, Munich, Poschingerstr. 5, Munich 81679, Bavaria, Germany; Department of Economics, University of Munich (LMU), Geschwister-Scholl-Platz 1, Munich 80539, Bavaria, Germany

**Keywords:** gender gap, preferences, personality traits, cohort replacement, age effects

## Abstract

Recent empirical literature has documented that men and women differ systematically in measures of patience, risk-taking, trust, and prosociality in many countries throughout the world. These gender gaps have been associated with variation in economic and social development. However, little is known about how these gender gaps evolve and whether they systematically vary across age cohorts. To examine this question, we analyze how within-country gender gaps in patience, risk-taking, trust, and prosociality have evolved. We compare gender gaps across country-period-cohort cells using two survey datasets covering 460,000 people in more than 100 countries. Our results document that gender gaps in patience, risk-taking, trust, and prosociality are smaller among younger cohorts, and suggest that this decline can be attributed to variation across birth cohorts rather than age groups. These findings challenge the conjecture that gender gaps widen as countries develop and instead point to an ongoing decline in the differences between men and women.

Significance statement Men and women have been found to differ systematically in economic and social preferences. These gender gaps are more pronounced in countries with higher levels of economic development and female empowerment. The remarkable stability of preferences over the life cycle and persistent gender differences in socioeconomic outcomes raise the question of how gender gaps in preferences evolve. Here, we investigate whether gender gaps in patience, risk-taking, trust, and prosociality differ across age cohorts. Using variation across age cohorts within countries, we document that gender gaps in these preferences are smaller among younger cohorts.

## Introduction

Attitudes toward time, risk, and social interactions drive people’s decision-making and determine economic outcomes. For instance, a person’s patience and willingness to take risks are associated with investment in stocks, occupational choices, and education (1, 2), while trust and prosociality are associated with labor market success (3).

Recent literature has developed survey measures of patience, risk-taking, trust, and prosociality that can be used to elicit information about the distribution of these attitudes in large, representative samples. This literature documents that patience, risk-taking, trust, and prosociality differ considerably throughout the world (4, 5). One striking finding of this literature is a pervasive gender gap: compared to men, women tend to be less patient, less willing to take risks, less reciprocal, more altruistic, and more trusting (4). This finding aligns with existing literature that documents systematic differences in preferences between men and women across various dimensions (6, 7) and shows that gender gaps in attitudes and preferences already emerge among adolescents (8, 9).

Although men’s and women’s socioeconomic outcomes have appreciably converged in high-income countries, the remaining gaps in wages and labor market activities appear remarkably persistent. The observed heterogeneity in preferences and attitudes between men and women might explain this persistence and impede further convergence in socioeconomic outcomes between men and women (10–12).

Growing evidence finds a positive association between economic development and gender gaps in patience, risk-taking, trust, and prosociality between countries: gender gaps are larger in richer and more gender-equal societies, where relaxed resource constraints allow men and women more scope to express gender-specific preferences (13, 14). These findings complement an extensive literature in personality and social psychology that has documented gender gaps in a large range of personality traits (15). This literature has reported a similar pattern of greater gender gaps in personality traits and occupational preferences in countries with higher levels of development and gender equality (16–19). This pattern suggests that gender gaps widen as countries develop, yet little is known about how gender gaps in patience, risk-taking, trust, and prosociality do evolve.

Here, we investigate global variation in within-country gender gaps in patience, risk-taking, trust, and prosociality (henceforth also referred to as “gender preference gaps”) across age cohorts. The analysis is motivated by two opposing hypotheses about whether gender gaps in preferences and attitudes originate from evolutionary or social mechanisms and how they evolve. Specifically, gender preference gaps might persist because of genetic predispositions that change little with socioeconomic development and could even widen if economic development facilitates the expression of gender-specific dispositions. In contrast, gender preference gaps might narrow as socioeconomic disparities between men and women shrink and traditional gender norms erode—especially among younger cohorts growing up under economically more favorable and gender-equal conditions than older cohorts. How gender preference gaps evolve across cohorts is thus primarily an empirical question.

The analysis is rooted in growing evidence suggesting that attitudes and preferences are formed early in life and remain fairly stable thereafter (20, 21). Empirical findings have documented that preferences exhibit very similar age patterns across birth cohorts (22, 23), indicating that preferences are shaped by age and cohort variation, with both sources of variation having distinct effects. While at the population level, preference heterogeneity is influenced by environmental factors related to geography, climate, and culture (24, 25), at the individual level, preference endowments are shaped by genetic factors, parental influences, and the social environment (26–28). Systematic cohort-level variation, such as experiencing severe recessions in the years of preference formation during adolescence, has also been shown to affect attitudes and preferences (29, 30). In view of the substantial improvements in economic living conditions over the past decades across large parts of the world, one might expect gender preference gaps to vary across age cohorts. Systematic variation in gender gaps across cohorts would be consistent with recent evidence showing that cultural change is driven by cohort replacement rather than by contemporaneous social influences and preference updating (31–33).

We study the evolution of gender gaps in patience, risk-taking, trust, and prosociality using two separate survey datasets. These surveys each provide representative country samples covering more than 90% of the world’s population and gross domestic product.

The first dataset is the Global Preferences Survey (GPS), which provides experimentally validated survey measures of time preference (patience), risk preference (risk-taking), generalized trust, altruism, and positive and negative reciprocity for 80,000 individuals from 76 countries. We follow previous work and consider economic preferences, trust, and a composite measure for prosociality (3, 34). A limitation for disentangling gender preference gaps across age groups and birth cohorts is that the GPS contains only a single survey wave from 2012, requiring restrictive structural identifying assumptions.

The second dataset is the World Values Survey (WVS), which contains data for 380,000 individuals from 106 countries and territories, observed across up to seven survey waves during 1981–2022. The WVS’s repeated cross-sections provide rich longitudinal information at the cohort level, which facilitates the identification of variation in gender preference gaps across birth cohorts and age groups. However, unlike the GPS, the WVS provides nonvalidated survey measures for patience, risk-taking, trust, and prosociality (unselfishness), which nevertheless have been frequently used in the literature as proxy measures for the respective preference dimensions (see, e.g. Ref. (35) for risk, (25) for patience, (36, 37) for trust, and (38) for prosociality; see also (39) for a comparison of measures for risk and time preferences).

While both datasets have limitations when used in isolation—one is constrained by data availability, the other by (lack of) validation of its measures—comparing their results lets us assess the robustness and generalizability of the estimated cohort variation in gender preference gaps under different identification assumptions and measures, thereby overcoming each dataset’s individual limitations.

Previous work on gender gaps in preferences and personality traits has relied on between-country comparisons (14, 16, 40). Here we make progress by leveraging within-country variation in gender preference gaps, complementing earlier between-country comparisons. By focusing on how gender preference gaps evolve within countries across age cohorts, our approach controls for systematic differences in the levels of patience, risk-taking, trust, and prosociality between men and women. Specifically, our approach accounts for country-specific and period-specific heterogeneity in gender preference gaps and eliminates, in various steps, country-, period-, gender-specific age profiles in the respective preference measures.

## Results

We investigate how gender gaps in survey measures of patience, risk-taking, trust, and prosociality have evolved within countries, measuring within-country gender preference gaps as the absolute difference in preference means between men and women. To construct these gaps, we aggregate respondents’ preference measures by country, survey period, and birth cohort (i.e. into country-period-cohort cells) for each gender and then calculate the absolute difference between the gender-specific cell means (for details on the survey items and variable construction, see Methods, Supplementary Appendix S.1, and (41)). We then analyze the extent to which gender gaps in patience, risk-taking, trust, and prosociality have increased, persisted, or decreased across age cohorts.

To gauge the importance of systematic age patterns in people’s preference measures (e.g. due to life-cycle stages or selective attrition), we compare gender gaps across birth cohorts before and after correcting the preference measures for systematic age profiles. Specifically, age-corrected preference measures are purged of country-, period-, gender-specific quadratic age profiles (see Methods for details and Figs. S.1 and S.2 for an illustration of age profiles in patience, risk-taking, trust, and prosociality by level of economic development and gender). Sample descriptions and summary statistics are reported in Supplementary Appendix S.2 (Tables S.1–S.4).

**Figure 1:**
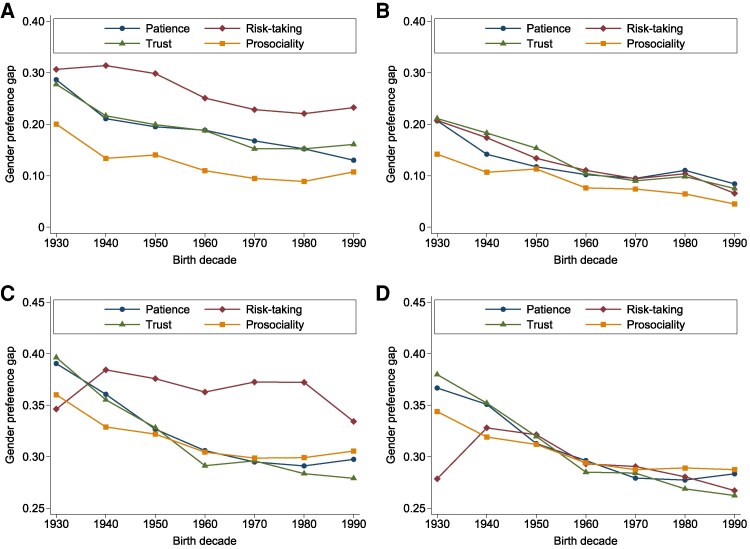
Unconditional gender gaps by birth decade. This figure shows the evolution of unconditional within-country gender gaps in patience, risk-taking, trust, and prosociality across birth decades. A and B) plot gender gaps in means of preference measures for data from the GPS, for preference gaps without age correction (A) and for preference gaps in age-corrected preference measures that have been purged of country-, gender-specific age profiles (B). C and D) plot gender gaps in means of for data from the WVS, without age correction (C) and for preference gaps in age-corrected preference measures that have been purged of country-, period-, gender-specific age profiles (D). All preference measures are standardized to mean 0 and SD 1 in the respective global samples such that gender preference gaps are expressed in SD of the corresponding preference measures (see Methods).

A descriptive analysis of the data reveals that within-country gender gaps in patience, risk-taking, trust, and prosociality are smaller among younger birth cohorts than among older ones. Figure 1A and C show gender gaps expressed in SD of the corresponding preference measures for different birth cohorts. The negative slopes of these lines indicate a common trend of declining gender preference gaps. This decline emerges in both the GPS and the WVS. The only exception is the gender gap in the risk-taking measure in the WVS, which follows a hump-shaped pattern across birth cohorts and, unlike the other preference measures, does not show an appreciable decline between 1930 and 1990 birth decades.

However, differences in the raw preference measures might mask differential age profiles in preferences: for instance due to metabolic or hormonal variation over the life cycle that differs between men and women and that affects various preferences in different ways. To account for such age-related variation and to isolate the evolution of gender gaps in preference, which is of main interest here, we apply an age correction that eliminates systematic country-, period-, and gender-specific age profiles before computing gender gaps in preferences. The differences in the corresponding age profiles suggest that preferences indeed exhibit systematic variation across different ages (see Figs. S.1 and S.2). The observed trends in age-corrected gender preference gaps across birth cohorts, depicted in Fig. 1B and D, show an even more homogeneous decline across birth cohorts, which is approximately linear. The gender preference gaps decline by about 0.05–0.15 SD of the corresponding preference measures between the 1930 and 1990 birth decades.

The unconditional gender preference gaps might still conceal systematic cross-country heterogeneity unrelated to cohort variation. To address this concern, we regress within-country gender gaps (with and without age correction) on birth decade, country fixed effects, and, in the longitudinal WVS data, country-period fixed effects. This approach allows us to estimate the global variation across birth cohorts in within-country gender preference gaps while accounting for heterogeneity in preferences driven by different country- and period-specific cultural, demographic, economic, institutional, and technological environments (see Methods). Standard errors are clustered at the country level. To ensure the estimates are ex-post representative at the country level, observations are weighted using individual-level sampling weights.

The estimation results consistently reveal a negative estimate for the birth decade coefficient, indicating that within-country gender gaps in patience, risk-taking, trust, and prosociality are smaller among later-born cohorts (Table 1). The estimated coefficients reflect the changes in gender preference gaps per birth decade, expressed in SD of the corresponding preference measures (equivalent to the slopes of linear trend lines in Fig. 1). We find shrinking gender preference gaps in both the GPS (Table 1A) and the WVS (Table 1B), regardless of whether preference measures were corrected for country-, period-, gender-specific age profiles. The estimated changes in gender preference gaps per birth decade are broadly similar across both datasets.

**Table 1:** Estimated changes in gender gaps per birth decade.

Gender gap in	Patience	Risk-taking	Trust	Prosociality
	(1)	(2)	(3)	(4)
A: Global Preferences Survey				
Dependent variable: Gender gaps without correction for age profiles in preferences				
Birth decade	− 0.021***	− 0.017***	− 0.018***	− 0.014***
	(0.004)	(0.005)	(0.005)	(0.003)
$R^{2}$	0.31	0.24	0.22	0.21
Clusters	76	76	76	76
Observations	513	513	512	513
Dependent variable: Gender gaps with correction for age profiles in preferences				
Birth decade	− 0.015***	− 0.022***	− 0.023***	− 0.015***
	(0.003)	(0.004)	(0.004)	(0.002)
$R^{2}$	0.32	0.29	0.29	0.29
Clusters	76	76	76	76
Observations	513	513	512	513
B: World Values Survey				
Dependent variable: Gender gaps without correction for age profiles in preferences				
Birth decade	− 0.012***	0.003	− 0.011***	− 0.012***
	(0.002)	(0.004)	(0.002)	(0.002)
$R^{2}$	0.23	0.29	0.24	0.24
Clusters	106	77	106	106
Observations	1,792	720	1,791	1,780
Dependent variable: Gender gaps with correction for age profiles in preferences				
Birth decade	− 0.012***	− 0.009**	− 0.008***	− 0.010***
	(0.002)	(0.003)	(0.002)	(0.002)
$R^{2}$	0.27	0.25	0.26	0.27
Clusters	106	77	106	106
Observations	1,792	720	1,791	1,780

Note: This table shows coefficient estimates from ordinary least squares regressions of within-country gender preference gaps on linear birth decade trends. The coefficients reflect the estimated changes in gender preference gaps per birth decade (i.e. cohort trends). Results are shown for 76 countries observed over 7 birth decades in the GPS (Panel A) and for 106 countries and territories observed over 7 birth decades and up to 7 survey waves during 1981–2022 in the WVS (Panel B). An observation is a within-country gender gap per birth decade. The dependent variable is the absolute difference in means of preferences between men and women (top of Panels A and B) or the absolute difference in means of age-corrected preferences between men and women (bottom of Panels A and B), where means are separately calculated for each gender within country-, period-, birth decade-specific cells. Age-corrected preference measures have been purged of country-, gender-specific age profiles in the cross-sectional data from the GPS and of country-, period-, gender-specific age profiles in the longitudinal data from the WVS. All preference measures are standardized to mean 0 and SD 1 in the respective global samples such that gender preference gaps are expressed in SD of the corresponding preference measures (see Methods). Specifications for the GPS include country fixed effects, whereas specifications for the WVS include country-period fixed effects. For a list of countries included in the GPS and WVS samples, see Tables S.1 and S.2. Standard errors are clustered at the country level and reported in parentheses. Asterisks indicate significance levels: * P<0.1; ** P<0.05; *** P<0.01.

**Figure 2:**
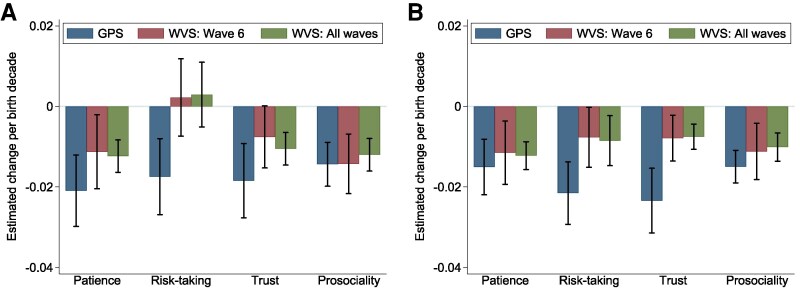
Estimated changes in gender gaps per birth decade: GPS vs. WVS. A) Gender gaps without age correction. This figure shows coefficient estimates and 95% CI from regressions of within-country gender preference gaps on linear birth decade trends. The coefficients reflect the estimated changes in gender preference gaps per birth decade (i.e. cohort trends). An observation is a within-country gender gap per birth decade. Data for the GPS comprise 76 countries observed over 7 birth decades. Data for the WVS comprise 106 countries observed over 7 birth decades and up to 7 survey waves during 1981–2022. The dependent variable is the absolute difference in means of preferences between men and women (A) and the absolute difference in means of age-corrected preferences between men and women (B). Age-corrected preference measures have been purged of country-, gender-specific age profiles in the cross-sectional data from the GPS and of country-, period-, gender-specific age profiles in the longitudinal data from the WVS. All preference measures are standardized to mean 0 and SD 1 in the respective global samples such that gender preference gaps are expressed in SD of the corresponding preference measures (see Methods). Specifications for the GPS include country fixed effects, whereas specifications for the WVS include country-period fixed effects. CI are based on SE that are clustered at the country level.

This similarity is reassuring in view of the additional identifying assumptions required for the GPS compared to the longitudinal WVS. The pattern holds regardless of whether we restrict the WVS sample to a cross-section elicited around the same time as the GPS (Wave 6) or include data from all 7 waves and country-period fixed effects (Fig. 2). For completeness, we also replicated the analysis for the subset of countries in the WVS for which at least two waves are available (Table S.5).

The estimated birth decade coefficient is qualitatively unchanged and quantitatively very similar when using data without applying sampling weights (Table S.6). Likewise, the results are very similar when additionally applying aggregate-level analytic weights that account for variation in the number of observations in gender and birth-decade cells (Table S.7).

Quantitatively, the results reveal a decline in gender preference gaps of ∼0.01–0.02 SD per birth decade (a decline of about 0.06–0.12 SD between the 1930 and 1990 birth decades). The estimated changes across birth cohorts imply that, on average, gender preference gaps have declined by 21.6% (ranging from 8.6 to 35.4%) over one generation between the 1950 and 1980 birth decades (Table S.8). To put this decline into perspective, the gender wage gap in the United States has decreased by about 50% between 1980 and 2010 (10).

Cohort heterogeneity accounts for an appreciable share of the variation in gender gaps in patience, risk-taking, trust, and prosociality. Specifically, it explains about 5–15% of the variation in gender preference gaps (Table S.9). Relative to cross-country heterogeneity, cohort heterogeneity explains about the same share of variation in the WVS and one-fourth to one-half as much in the GPS.

Our finding of declining gender preference gaps across birth cohorts also holds when we apply highly restrictive age corrections that use higher-order polynomials or nonparametric age bins to purge preference measures of country-, period-, gender-specific age profiles in order to account for nonmonotonic and discontinuous age patterns (Fig. S.3).

We note two interesting patterns in this context. First, more flexible specifications of the age correction lead to smaller estimates of the cohort variation in the cross-sectional data from the GPS. By contrast, the estimated cohort variation is essentially unaffected by how we specify the age correction in the longitudinal data from the WVS. Second, the estimated cohort variation in the GPS and the WVS is very similar in size for the most flexible (nonparametric) specification of the age correction. These patterns reflect differences in the potential for identifying cohort variation in cross-sectional versus longitudinal data (see Methods). In cross-sectional data, the identification of variation across birth cohorts requires an assumption regarding the age profiles in the preference measures because more flexible specifications of the age correction mechanically absorb cohort variation. In longitudinal data, such an assumption is unnecessary because age and cohort variation are observed separately from one another.

Additional robustness checks support the finding of declining within-country gender gaps in patience, risk-taking, trust, and prosociality. The analyses for the WVS deliver similar results when assuming stable age profiles in these attitudes and applying the age correction for all waves jointly rather than for each wave separately (Table S.10), and when accounting for cohort-specific age profiles (Table S.11). Finally, the results are also robust to using country–period–birth year cells rather than country–period–birth decade cells. Fitting a model that uses birth year data, we can simultaneously incorporate linear variation across birth decades and arbitrary age profiles while controlling for country-period fixed effects. The corresponding results are quantitatively similar to the baseline results and reveal a decline in gender gaps across birth cohorts in patience, risk-taking, trust, and prosociality, conditional on age group variation (Table S.12).

Taken together, the comparison across different specifications of age profiles in the WVS reveals no systematic age variation in the age-corrected measures and supports the finding of a decline in gender gaps in patience, risk-taking, trust, and prosociality across birth cohorts. These findings are consistent with existing literature suggesting that preferences exhibit very similar life-cycle patterns across birth cohorts (23). Moreover, the similarity of our results for the GPS and the WVS further supports the interpretation of our results in terms of a negative global trend in gender preference gaps across birth cohorts.

Additional analysis reveals that the dynamics in gender gaps in patience, risk-taking, trust, and prosociality are not driven by between-country heterogeneity in the levels of economic development and gender equality. Within-country gender preference gaps are only weakly and unsystematically associated with between-country heterogeneity in income per capita and gender equality (Tables S.13–S.16). Moreover, the results also document a decline in gender preference gaps across birth cohorts when controlling for income per capita and gender equality.

**Figure 3:**
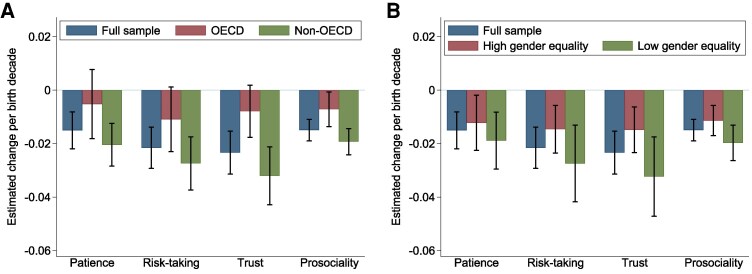
Estimated changes in gender gaps per birth decade by economic development and gender equality. This figure shows coefficient estimates and 95% CI from regressions of within-country gender preference gaps in the GPS on linear birth decade trends. The coefficients reflect the estimated changes in gender preference gaps per birth decade (i.e. cohort trends). The data comprise 76 countries observed over 7 birth decades. An observation is a within-country gender gap per birth decade. The dependent variable is the absolute difference in means of age-corrected preferences between men and women. All preference measures are standardized to mean 0 and SD 1 in the respective global samples such that gender preference gaps are expressed in SD of the corresponding preference measures (see Methods). The figure shows heterogeneity in the estimated changes in gender preference gaps per birth decade by level of economic development in terms of OECD member status (A) and by level of gender equality (B). A country is defined as gender-equal if its gender parity score in the World Economic Forum’s gender parity index (42) exceeds the median. All specifications include country fixed effects. CI are based on SE that are clustered at the country level.

However, the size of the cohort dynamics in gender preference gaps can vary across country samples. One dimension of heterogeneity refers to differences with respect to economic development and gender equality. When we stratify our sample by Organisation for Economic Co-operation and Development (OECD) member status and a gender parity index, gender gaps in patience, risk-taking, trust, and prosociality decline less in more developed and emancipated countries. In OECD member states—which are predominantly Western, educated, industrialized, rich, and democratic (43)—the estimated changes in gender preference gaps in the GPS per birth decade are comparatively smaller and only marginally (or not at all) significant; in non-OECD countries, they are comparatively larger and highly significant (Fig. 3A). Gender gaps also decline less in more gender-equal societies (Fig. 3B). Similar results hold for the WVS (Fig. S.4), and for gender gaps without age correction (Figs. S.5 and S.6). Focusing on effect heterogeneity with respect to income alone delivers relatively larger estimates for the decline in gender preference gaps across cohorts for low-income countries and lower middle-income countries (Fig. S.7).

The stronger convergence in gender preference gaps among economically less developed countries might reflect variation in gender differences across societies. Countries that exhibit little or no gender difference in preferences among older cohorts have less scope for subsequent convergence than countries with large gender differences. We explored this conjecture about heterogeneity in the scope for convergence in the gender preference gap by stratifying the sample into four equally sized sub-samples depending on the level of the gender preference gap in the cohort born during the 1940s. We found that countries with larger initial gender preference gaps converge faster than those with smaller initial gender preference gaps (Fig. S.8).

Finally, further analyses reveal that the decline in gender preference gaps across birth cohorts not only pertains to differences in preference means but also to differences in the variability of preferences (SD) between men and women (Fig. S.9). This finding complements decreasing gender gaps in preference means and suggests that the shapes of the preference distributions of men and women are also more similar among later-born cohorts.

## Discussion

Our results document that gender gaps in patience, risk-taking, trust, and prosociality are smaller among younger cohorts. This pattern emerges in two separate datasets, which are each representative of more than 90% of the world’s population and gross domestic product, pertains to gender gaps in both levels and variability, and reflects convergence in the distributions of the respective preference measures. The results indicate that, on average, the gaps in measures of patience, risk-taking, trust, and prosociality between men and women born in the 1980s are about 20% smaller than among men and women born in the 1950s.

We interpret our evidence of shrinking gender preference gaps as a narrowing of the differences in patience, risk-taking, trust, and prosociality between men and women among younger birth cohorts. This interpretation rests on the assumption that age and cohort effects in these preference measures are separable, which is common in the psychology and economics literature and consistent with recent evidence (23). Without this assumption, it is fundamentally impossible to disentangle age from cohort patterns—an issue that applies to related contributions as well.

The estimated coefficients for the uncorrected measures of patience, risk-taking, trust, and prosociality might capture variation across both age groups and birth cohorts. In contrast, the estimated coefficients for the age-corrected preference measures reflect changes in gender preference gaps after country-, period-, gender-specific age variation has been removed. If the age correction adequately removes the age profiles in the respective preference measures, the estimated changes in gender gaps reflect cohort effects (for a technical discussion, see Methods).

Our results document remarkably similar cohort variation in gender preference gaps, both with and without age corrections, across two distinct datasets. In addition, the data reveal age profiles that are distinct across preferences, but similar across data sets for a given preference domain. This consistency provides indirect support for the plausibility of the separability assumption and bolsters our finding of smaller within-country gender differences in patience, risk-taking, trust, and prosociality among the younger cohorts. In particular, our evidence of the stability of the estimated changes in gender gaps across birth cohorts in the WVS, using both parametric and nonparametric age corrections, supports the plausibility of the cohort interpretation (see Fig. S.3b). However, regardless of the exact interpretation as variation across age groups or birth cohorts, our results contribute evidence of smaller gender preference gaps among younger cohorts.

Recent evidence has pointed to greater variability in preferences among men than among women (44, 45). This finding suggests the importance of considering within-gender variability of preference measures for the analysis of gender differences in behavior and outcomes. Our result of shrinking gender preference gaps in both means and SD across birth cohorts indicates convergence in the preference distributions of men and women. In addition, recent work has emphasized the distinction between gender preference gaps and believed gender preference gaps (46). Our findings instead relate to observed gender preference gaps.

To examine gender norms more directly, we considered survey information about attitudes toward gender roles in the WVS. Specifically, we use reported approval of whether “when jobs are scarce, men should have more right to work than women” to examine whether gender gaps in such norms exhibit similar cohort or cross-country patterns as the preference gaps documented in the main analysis. Empirical results show that gender gaps in norms measured this way are indeed smaller among younger cohorts when accounting for systematic age profiles in norms (Table S.17). When replicating the main analysis controlling for differences in gender norms across cohorts, we find that greater differences in gender norms are associated with greater gender preference gaps, with the estimated cohort trends remaining significant (Table S.18). A more detailed exploration of norms and beliefs using a similar methodology constitutes an interesting avenue for future research.

Previous literature has proposed alternative explanations for gender differences in personality traits. These include intergenerational transmission (27, 47), evolutionary theories, and theories of perceived gender roles as the result of socialization and differentials in social power (19, 48). According to evolutionary theories, gender gaps are innate and reflect the adaptation of preferences and traits to specific geographic, climatic, and cultural environments in the ancestral past and their selective transmission across generations (e.g. (49–51)). Supporting this perspective, cross-country differences in biological, cultural, and economic factors have been found to explain gender differences in personality (52).

*Prima facie*, the finding of a decline in gender preference gaps might appear to contradict recent evidence that has found gender preference gaps to be wider in more developed and gender-equal economies (14, 16, 53). An explanation for this pattern is that by relaxing material and social constraints characteristic of agricultural societies, economic development could allow for more gender-specific expression of preferences. The narrowing of gender gaps in patience, risk-taking, trust, and prosociality found here reflects within-country cohort variation found worldwide and is consistent with narrowing observed in other socioeconomic outcomes. At the same time, the observation of smaller gender preference gaps for later-born cohorts is compatible with systematic variation in the levels of gender preference gaps across countries, which are absorbed by the estimation framework.

Additional analyses indicate that gender preference gaps narrow more rapidly in low-income countries than in high-income countries, suggesting the within-country dynamics differ from the between-country patterns that previous literature has used to document larger gender preference gaps in high-income countries. In fact, recent work that investigates gender gaps in honesty both within and across countries shows that gender gaps primarily emerge in more affluent Western societies and that they narrow as countries develop economically (54). These findings also point toward vertical transmission of preferences across generations rather than a resource-based explanation. Our results complement these findings by documenting a decline in gender preference gaps across cohorts. This decline is consistent with preference formation early in life being influenced by intergenerational and oblique transmission of preferences (27) and points to cultural change through cohort replacement (see, e.g. Refs. (31–33)).

According to theories of the social determination of gender roles, gender gaps in personality reflect the specialization and division of labor, societal roles, and social power by gender (53). The influence of time-invariant country-specific heterogeneity in cultural norms or factors that are related to gender gaps is absorbed by country fixed effects in the empirical framework. Additional analysis with explicit controls for various factors whose relevance has been shown in previous work on gender roles and preferences (e.g. (25, 49)) does not reveal evidence for particular factors driving the gender gap in a specific preference dimension, while leaving the main finding of smaller differences among later-born cohorts unaffected (Tables S.19 and S.20).

Of course, historically determined psychological patterns, social norms, and gender roles may change with socioeconomic conditions and institutions (55, 56). For instance, with labor markets’ diminishing reliance on physical and biological factors and increasing gender equality, gender differences in personality tend to weaken over time (16, 40). These observations raise the question of whether there is heterogeneity in the convergence of preferences between men and women. Additional results confirm the finding of a universal decline in gender preference gaps across cohorts but point to a slightly weaker convergence in countries where gender norms are more deeply rooted, as proxied by plow use or absolute latitude (Tables S.21 and S.22).

Our evidence suggests that social rather than innate factors are the primary drivers of gender gaps in patience, risk-taking, trust, and prosociality insofar as the mechanisms giving rise to the gender gaps in these dimensions parallel those for other personality traits. If preferences are formed during the impressionable years of adolescence and remain fairly stable thereafter, as suggested by the prevalent view in the literature (20, 21, 57), then the observed narrowing of gender preference gaps across birth cohorts might result from transformative processes related to globalization, technological progress, sectoral transformation, and institutional change. These changes expose men and women to increasingly similar environments regarding information, lifetime experiences, and socioeconomic conditions during the ages of preference formation. Cohort variation might thus reflect a convergence in macroeconomic or other contextual conditions during the impressionable years of adolescence, rather than long-run evolutionary factors.

The finding of declining gender gaps in patience, risk-taking, trust, and prosociality across birth cohorts also complements previous literature that has found greater gender preference gaps in high-income countries and countries with greater gender equality (13, 14). Recent evidence suggests this finding disappears when considering variation within cultural clusters of countries (58). By accounting for systematic between-country differences in gender preference gaps via country fixed effects, we document a decline in preference gaps across birth cohorts. Gender preference gaps converge faster in less developed and more gender-unequal countries and more slowly in richer and more gender-equal societies. Although the estimated cohort profiles are not in all cases statistically significant, our results qualify a common conclusion from cross-country studies: greater gender preference gaps in more developed countries do not imply that gender gaps in economic and social preferences widen as countries develop. Instead, we demonstrate that greater gender gaps in patience, risk-taking, trust, and prosociality in richer and more gender-equal countries can occur alongside a decline in preferences between men and women across birth cohorts in those countries.

In an increasingly connected world, cohort replacement predicts further convergence in gender preference gaps. Recent evidence supports this view, showing that economic and social preferences have also become more similar among later-born cohorts worldwide (59). The evidence of a decline in within-country gender preference gaps presented in this article constitutes an important facet of this preference convergence: despite greater gender differentiation in preference levels among Western, educated, industrialized, rich, and democratic countries (13, 14), gender preference gaps tend to shrink as societies develop. Our evidence of narrowing gender preference gaps across birth cohorts aligns with the convergence in socioeconomic outcomes between men and women observed in many countries in recent years and suggests this trend might continue.

## Methods

### Data and samples

The analysis is based on two independent datasets, covering countries from all continents and at various stages of economic development. Our first dataset is the GPS, which includes preference measures for ∼80,000 individuals in 76 countries (4). Conducted as part of the 2012 Wave of the Gallup World Poll, this survey contains a module of preference items for time preference (patience), risk preference (risk-taking), generalized trust, and prosociality (comprising the averages of the measures of altruism, positive reciprocity, and negative reciprocity). The behavioral reliability of these preference measures was validated in incentivized laboratory experiments (41). Additional studies further validated the same or very similar survey items in various populations across high- and low-income countries (2, 60–64). The GPS contains only a single survey wave, which limits the variation available for identifying gender preference gaps across birth cohorts.

To address this issue, we alternatively use survey measures from the WVS, which comprises preference measures for ∼380,000 individuals across 106 countries and territories, collected over up to 7 waves during 1981–2022 (65). The preference measures in the WVS were not validated and differ from those in the GPS, except for the trust measure, which is identical in both datasets. The repeated cross-sections in the WVS provide longitudinal variation in preferences, allowing us to account for changes in gender preference gaps over time and across birth cohorts.

Tables S.1 and S.2 list the countries included in the two datasets, and Tables S.3 and S.4 report descriptive statistics.

### Preference measures

The preference measures in the GPS are based on survey items involving hypothetical choice experiments and subjective self-assessments in the context of intertemporal choice (patience), willingness to take risks (risk-taking), trust, altruism (the willingness to incur a cost in order to benefit others without expecting anything in return), positive reciprocity (the willingness to reward kind behavior by others), and negative reciprocity (the willingness to punish unkind behavior by others). The specific survey items were selected through a validation procedure that identified items from a long list of candidate questions based on their predictive power for incentivized choices regarding the respective preference dimensions in laboratory experiments. The resulting six preference measures were constructed as composites of the respective survey items (for details, see (41)).

The preference measures in the WVS are nonvalidated proxy measures. They are based on responses to questions about desirable qualities of children and personal values. Specifically, these measures capture whether thrift is a desirable quality of children (capturing patience), how important it is to experience adventures and take risks (capturing the willingness to take risks), and whether not being selfish (unselfishness) is a desirable quality of children (capturing prosociality). Although they are less direct proxies for preferences than the measures in the GPS, the measures of the different preference dimensions in both datasets are systematically correlated (see Ref. (4)).

While both the GPS and the WVS have limitations when used in isolation (the former is constrained by data availability, the latter by lack of validation of its measures), conducting the analysis for both datasets and comparing the results sheds light on the robustness and generalizability of cohort variation in gender gaps under different identification assumptions and measures.

All preference measures are standardized to mean 0 and SD 1 in the respective global samples (for details on the survey items, see Supplementary Appendix S.1.

### Statistical analysis

The measure for a particular preference of a male respondent *r* in country *i* who is a member of birth cohort *t* is denoted as $P_{r,i,t}^{m}$. The corresponding preference measure of a female respondent $r^{\prime }$ is $P_{r^{\prime },i,t}^{f}$. Preference measures for each gender are aggregated into country-period-cohort cells, where the corresponding means for men and women are given by $\mu_{i,t}^{m}=\frac{1}{n_{i,t}^{m}}\sum_{r=1}^{n_{i,t}^{m}}P_{r,i,t}^{m}$ and $\mu_{i,t}^{f}=\frac{1}{n_{i,t}^{f}}\sum_{r^{\prime }=1}^{n_{i,t}^{f}}P_{r,i,t}^{f}$ and where $n_{i,t}^{m}$ and $n_{i,t}^{f}$ denote the respective counts of male and female respondents of birth cohort *t* in country *i*. The GPS represents one cross-section, such that all respondents are observed in the same period. In contrast, preference measures in the WVS are elicited across up to 7 survey waves and separately aggregated by country, birth cohort, and gender for each survey wave (the notation for survey waves is suppressed for convenience). The average gender preference gap $H_{i,t}$ is then given by the absolute difference in country-, period-, cohort-specific gender preference means:


$$H_{i,t}=\left|\mu_{i,t}^{m}-\mu_{i,t}^{f}\right|.$$

For the construction of country-, period-, cohort-specific means, we use the individual-level sampling weights provided by the Gallup World Poll and the WVS. These weights ensure that the respective means are representative of the population of a given country.

To account for systematic gender-specific variation in preferences across age groups that might influence the measures of the gender preference gaps, alternative age-corrected measures are constructed using the residuals of regressions that regress respondents’ preferences on country-, gender-specific age patterns. Specifically,


$${\tilde{P}}_{r,i,t}^{g}=P_{r,i,t}^{g}-\hat{F}\left({\mathrm{Age}}_{r,i,t}\right),$$

where g=m,f denotes gender (male, female), ${\mathrm{Age}}_{r,i,t}$ denotes respondents’ age, and $\hat{F}({\mathrm{Age}}_{r,i,t})$ denotes estimates of parametric or nonparametric age patterns. In the baseline specification, the age pattern is assumed quadratic such that $\hat{F}({\mathrm{Age}}_{r,i,t})={\hat{\gamma }}_{0,i}^{g}+{\hat{\gamma }}_{1,i}^{g}\;{\mathrm{Age}}_{r,i,t}+{\hat{\gamma }}_{2,i}^{g}\;{\mathrm{Age}}_{r,i,t}^{2}$ with ${\hat{\gamma }}_{0,i}^{g}$, ${\hat{\gamma }}_{1,i}^{g}$, and ${\hat{\gamma }}_{2,i}^{g}$ representing country-, gender-specific coefficient estimates of the respective age profiles. Additional analysis uses alternative specifications, such as cubic or quartic age trends or a nonparametric specification that allows for a flexible age profile based on 10-year age bins. This age correction is conducted once for the GPS. For the WVS, we analyze the data using two different age corrections: (i) assuming a stable gender-specific age profile for a country across all survey waves and (ii) allowing for country-gender-specific age variation for each survey wave. These regressions also use the individual-level sampling weights provided by the Gallup World Poll and the WVS, respectively, to ensure representativeness.

Age-corrected measures of the gender gaps in each preference, ${\tilde{H}}_{i,t}$, are then constructed analogously based on the residualized individual preference measures. More restrictive age corrections are used in robustness analyses where we estimate country-, period-, gender-specific age profiles parametrically with cubic and quartic age trends or nonparametrically with 10-year age bins, and then purge preference measures of the corresponding age profiles. Country-, period-, cohort-specific means of age-corrected preference measures are computed using the individual-level sampling weights provided by the Gallup World Poll and the WVS.

The analysis of within-country gender preference gaps is based on the empirical model


$$H_{i,t(,p)}=\tau D(t)+\zeta X_{i,t}+\xi_{i(,p)}+\epsilon_{i,t(,p)},$$

where D(t) denotes a birth decade and *τ* measures variation in gender gaps across age cohorts; $X_{i,t}$ denotes additional controls, such as log income per capita, that might affect gender preference gaps; and $\xi_{i(,p)}$ denotes country-period fixed effects, which account for systematic variation in gender preference gaps by country in the cross-sectional GPS and for systematic variation in gender preference gaps by country-period in the longitudinal WVS. The residual $\epsilon_{i,t(,p)}$ captures idiosyncratic variation in gender preference gaps at the country level.

The analysis of cohort patterns in gender preference gaps is based on the conceptual assumption that age and cohort patterns in preference measures are separable from one another. This assumption is common in the literature on personality traits and preferences and is supported by recent evidence documenting that age profiles in preferences are stable across cohorts (23).

The identification of cohort patterns requires additional assumptions that vary across different specifications of the empirical model. Specifically, we analyze the variation in gender preference gaps at the country-cohort level with two approaches. The first approach constructs gender preference gaps for country-cohort cells and uses these gaps as the dependent variable. The additional assumption required for this first approach is that men and women within a country have identical age patterns that are constant across cohorts. If that is the case, the estimation results isolate cohort variation in gender gaps because parallel age profiles are removed when calculating gender preference gaps for each country-cohort cell.

Our second approach relaxes the assumption of identical age patterns in preferences of men and women in a country, allowing for age profiles to systematically differ between men and women. To this end, the analysis uses of age-corrected measures that have been purged of quadratic country-gender-specific age profiles. This approach explicitly accounts for heterogeneous age profiles of men and women from the same country before computing gender preference gaps and estimating common cohort variation in these gaps. The additional assumption required for this second approach is that the age correction adequately accounts for any age patterns in the preference measures.

We probe the plausibility of this assumption by applying alternative, more restrictive age corrections that purge the preference measures of cubic, quartic, and nonparametric country-, period-, gender-specific age patterns. In addition, the use of repeated observations for the same countries over time in the WVS allows us to construct a pseudo-panel at the level of country-cohort-gender cells. By observing the same birth cohorts at different ages within a country, and the same age groups across birth cohorts, one can disentangle linear variation across age groups and birth cohorts while accounting for systematic variation across time periods. This data structure allows us to examine the sensitivity of the estimated patterns in gender preference gaps with respect to the specific functional form of our age correction.

## Supplementary Material

pgag255_Supplementary_Data

## Data Availability

The primary data that support the findings of this study are publicly available from the websites of the Global Preferences Survey (https://gps.econ.uni-bonn.de/home) and of the World Values Surveys (https://www.worldvaluessurvey.org). The code to reproduce the data and replicate the analysis, including all tables and figures, are accessible on Harvard Dataverse ((67), doi.org/10.7910/DVN/VBZPSC).

## Preprints

An earlier version of this manuscript was posted on a preprint ((66), https://www.econstor.eu/bitstream/10419/331638/1/cesifo1_wp12173.pdf).
