# Supplementary material for: Gender gaps in patience, risk-taking, trust, and prosociality are smaller among younger cohorts
Source: PNAS Nexus. 2026 Aug 11;5(8):pgag255. doi: 10.1093/pnasnexus/pgag255 (PMC13463103; doi:10.1093/pnasnexus/pgag255)
Supplement: Supplementary file 1 [file pgag255_supplementary_data.pdf]

SUPPLEMENTARY APPENDIX
Gender Gaps in Patience, Risk-Taking, Trust, and Prosociality Are Smaller among Younger Cohorts

Rainer Kotschy[1] and Uwe Sunde[2]

[1]NBER, Harvard University, and CESifo

[2]LMU Munich, CESifo, CEPR, and IZA

Contents

S.1 Supplementary Appendix: Preference Measures **S.1**

S.2 Supplementary Appendix: Additional Figures and Tables **S.6**

List of Figures

S.1 Age profiles by economic development and gender: Global Preferences Survey S.9
S.2 Age profiles by economic development and gender: World Values Survey . . . S.10
S.3 Estimated changes in gender gaps per birth decade: Alternative age corrections S.11
S.4 Estimated changes in gender gaps per birth decade by economic development and gender equality . . . S.11
S.5 Estimated changes in gender gaps per birth decade by economic development and gender equality: Results without correction for age profiles in preference measures . . . S.12
S.6 Estimated changes in gender gaps per birth decade by economic development and gender equality: Results without correction for age profiles in preference measures . . . S.12
S.7 Estimated changes in gender gaps per birth decade by income level . . . S.13
S.8 Estimated changes in gender gaps per birth decade by initial gender gaps . . . S.13
S.9 Estimated changes in gender variability gaps per birth decade: Global Preferences Survey vs. World Values Survey . . . S.14

List of Tables

S.1 List of countries in sample: Global Preferences Survey S.6
S.2 List of countries in sample: World Values Survey S.7
S.3 Descriptive statistics S.8
S.4 Descriptive statistics by OECD member status S.8
S.5 Estimated changes in gender gaps per birth decade: Only countries with two or more waves S.15
S.6 Estimated changes in gender gaps per birth decade: No weighting during age correction and data aggregation S.16
S.7 Estimated changes in gender gaps per birth decade: Results weighted by cell size S.17
S.8 Estimated decline in gender gaps between 1950 and 1980 birth decades S.18
S.9 Variation in gender gaps explained by cohort fixed effects and country fixed effects S.18
S.10 Estimated changes in gender gaps per birth decade: Stable age profiles S.19
S.11 Estimated changes in gender gaps per birth decade: Cohort-specific age profiles S.19
S.12 Estimated changes in gender gaps by age and cohort S.20
S.13 Gender gaps without age correction: Global Preferences Survey S.21
S.14 Gender gaps with age correction: Global Preferences Survey S.22
S.15 Gender gaps without age correction: World Values Survey S.23
S.16 Gender gaps with age correction: World Values Survey S.24
S.17 Estimated changes in gender gaps of employment preferences per birth decade . S.25
S.18 Estimated changes in gender gaps per birth decade: Accounting for gender attitudes S.26
S.19 Estimated changes in gender gaps per birth decade: Accounting for historical determinants of preference formation in the Global Preferences Survey S.27
S.20 Estimated changes in gender gaps per birth decade: Accounting for historical determinants of preference formation in the World Values Survey S.28
S.21 Estimated changes in gender gaps per birth decade: Birth-decade trends interacted with select determinants of gender gaps in the Global Preferences Survey S.29
S.22 Estimated changes in gender gaps per birth decade: Birth-decade trends interacted with select determinants of gender gaps in the World Values Survey . . . 30

S.1 Supplementary Appendix: Preference Measures

Global Preferences Survey

The Global Preferences Survey provides individual measures for six preferences: time preference, risk preference, trust, altruism, positive reciprocity, and negative reciprocity. These measures are constructed from responses to one or several survey items and standardized to have mean zero and variance one. We describe the detailed content of the respective survey items subsequently.

Patience. The patience measure (time preference) is constructed by combining a qualitative and a quantitative survey item. The qualitative survey item asked respondents to assess their willingness to forego something beneficial today in order to benefit more from that in the future on a scale from 0 (unwilling) to 10 (very willing). The exact wording was as follows: "How willing are you to give up something that is beneficial for you today in order to benefit more from that in the future? Please use a scale from 0 to 10, where 0 means "completely unwilling" and a 10 means you are "very willing". You can also use any numbers between 0 and 10 to indicate where you fall on the scale, like 0, 1, 2, 3, 4, 5, 6, 7, 8, 9, 10." The quantitative item confronted respondents with five interdependent choices between an immediate payment and varying future payments with a 12-month delay, assuming no inflation. The exact wording was as follows: "Suppose you were given the choice between receiving a payment today or a payment in 12 months. We will now present to you five situations. The payment today is the same in each of these situations. The payment in 12 months is different in every situation. For each of these situations we would like to know which you would choose. Please assume there is no inflation, i.e, future prices are the same as today's prices. Please consider the following: Would you rather receive 100 Euro today or x Euro in 12 months?" The precise sequence of future amounts was dependent on response behavior following a staircase logic (see **?**, for details). Payments were adjusted to average income in a country to establish comparability. Both items were combined into a single composite measure based on the validation results for the survey items (see **?**, for details).

Risk-taking. The risk-taking measure (risk preference) is constructed by combining a qualitative and a quantitative survey item. The qualitative item asked respondents to assess their willingness to take risks on a scale from 0 (unwilling) to 10 (very willing). The exact wording

was as follows: “Please tell me, in general, how willing or unwilling you are to take risks. Please use a scale from 0 to 10, where 0 means “completely unwilling to take risks” and a 10 means you are “very willing”. You can also use any numbers between 0 and 10 to indicate where you fall on the scale, like 0, 1, 2, 3, 4, 5, 6, 7, 8, 9, 10.” The quantitative item presented respondents with a sequence of five hypothetical interdependent choices between a varying safe payment and a binary lottery offering either a positive payment or no payment, each with 50 percent probability. The exact wording was as follows: “Please imagine the following situation. You can choose between a sure payment of a particular amount of money, or a draw, where you would have an equal chance of getting amount x or getting nothing. We will present to you five different situations. What would you prefer: a draw with a 50 percent chance of receiving amount x, and the same 50 percent chance of receiving nothing, or the amount of y as a sure payment?” The precise sequence of the sure payment y was dependent on response behavior following a staircase logic (see **?**, for details). To ensure comparability across countries, payments were adjusted based on purchasing power parity and relative to median household income for each country. Both items were combined into a single composite measure based on the validation results for the survey items (see **?**, for details).

Trust. The trust measure (social preference, belief) is based on respondents’ agreement with the statement, “As long as I am not convinced otherwise, I assume that people have only the best intentions.” Respondents indicated their agreement on a scale from 0 (does not apply) to 10 (fully applies).

Prosociality. We constructed a prosociality measure (social preference) by combining the survey measures for altruism, positive reciprocity, and negative reciprocity. We calculated the arithmetic mean of the standardized values for altruism, positive reciprocity, and negative reciprocity and standardized that measure to have mean zero and standard deviation one.

The altruism measure is constructed by combining a qualitative and a quantitative survey item. The qualitative item asked respondents to assess their willingness to give to good causes without expecting anything in return on a scale from 0 (unwilling) to 10 (very willing). The exact wording was as follows: “How willing are you to give to good causes without expecting anything in return? Please use a scale from 0 to 10, where 0 means “completely unwilling to take risks” and a 10 means you are “very willing”. You can also use any numbers between 0 and 10 to indicate where you fall on the scale, like 0, 1, 2, 3, 4, 5, 6, 7, 8, 9, 10.” The quantitative

item asked respondents to imagine an unexpected windfall gain of €1,000 and indicate how much of that win they would donate to charity. The exact wording was as follows: "Imagine the following situation: Today you unexpectedly received 1,000 Euro. How much of this amount would you donate to a good cause? (Values between 0 and 1000 are allowed.)" To ensure comparability across countries, payments were adjusted based on purchasing power parity and relative to median household income for each country. Both items were combined into a single composite measure based on the validation of the survey items (see **?**, for details).

The positive reciprocity measure is constructed by combining a qualitative and a quantitative survey item. The qualitative survey item asked respondents to assess their willingness to return a favor, measured on a scale from 0 (not willing) to 10 (very willing). The exact wording was as follows: "When someone does me a favor I am willing to return it. Please use a scale from 0 to 10, where 0 means "completely unwilling to take risks" and a 10 means you are "very willing". You can also use any numbers between 0 and 10 to indicate where you fall on the scale, like 0, 1, 2, 3, 4, 5, 6, 7, 8, 9, 10." The quantitative item asked respondents to imagine receiving help with directions and to indicate, on a scale from €0 to €30 (in increments of €5), how much they would give as a thank-you gift. The exact wording was as follows: "Please think about what you would do in the following situation. You are in an area you are not familiar with, and you realize you lost your way. You ask a stranger for directions. The stranger offers to take you to your destination. Helping you costs the stranger about 20 Euro in total. However, the stranger says he or she does not want any money from you. You have six presents with you. The cheapest present costs 5 Euro, the most expensive one costs 30 Euro. Do you give one of the presents to the stranger as a thank-you-gift? If so, which present do you give to the stranger? No present / The present worth 5 / 10 / 15 / 20 / 25 / 30 Euro." To ensure comparability across countries, payments were adjusted based on purchasing power parity and relative to median household income for each country. Both items were combined into a single composite measure based on the validation of the survey items (see **?**, for details).

The negative reciprocity measure is constructed by combining three qualitative survey items. These items asked respondents to assess on a scale from 0 (not willing) to 10 (very willing) their willingness to (i) take revenge in case of being treated very unjustly, even at a cost; he exact wording of the question was as follows: "If I am treated very unjustly, I will take revenge at the first occasion, even if there is a cost to do so." (ii) to punish someone who treated the respondent unfairly, even at a cost; the exact wording of the question was as follows: "How willing are you

to punish someone who treats you unfairly, even if there may be costs for you?" and (iii) to punish someone who treated others unfairly, even at a cost; the exact wording of the question was as follows: "How willing are you to punish someone who treats others unfairly, even if there may be costs for you?". The items were combined into a single composite measure based on the validation of the survey items (see **?**, for details).

Order of Questions. The order of survey items was as follows: Willingness to take risks (qualitative item), patience (qualitative item), negative reciprocity (qualitative items for punishment of unfair behavior towards respondent, towards others), positive reciprocity (qualitative item), negative reciprocity (qualitative item for revenge), trust, willingness to take risks (quantitative item), positive reciprocity (quantitative item), altruism (quantitative item), patience (quantitative item). The questionnaire with all questions and their exact order are publicly available at the website of the Global Preferences Survey (`https://gps.econ.uni-bonn.de/home`).

World Values Survey

The World Values Survey contains several items that are frequently used as proxy measures for patience, risk-taking, trust, and prosociality. These measures are standardized to have mean zero and variance one. We describe the detailed content of the respective survey items subsequently.

Patience. The patience measure (time preference) is based on responses to a question about qualities that children can be encouraged to learn at home. Respondents are asked which of up to five qualities they consider to be especially important. The exact wording was as follows: "Here is a list of qualities that children can be encouraged to learn at home. Which, if any, do you consider to be especially important? Please choose up to five! (Code five mentions at the maximum): Thrift, saving money and things". The respective measure takes value 1 if respondents mention thrift (saving money and things), and 0 otherwise.

Risk-taking. The risk-taking measure (risk preference) is based on responses to a question about how important it is to the respondent to experience adventure and taking risks on a scale from 1 (very much like me) to 6 (not at all like me). The exact wording was as follows: "Now I will briefly describe some people. Using this card, would you please indicate for each description whether that person is very much like you, like you, somewhat like you, not like you, or not at all like you? Adventure and taking risks are important to this person; to have an exciting life".

Trust. The trust measure (social preference, belief) is based on respondents' agreement with the statement, "I see myself as someone who is generally trusting." Respondents indicated their agreement on a scale from 1 (agree strongly) to 5 (disagree strongly).

Prosociality. The prosociality measure (social preference) is based on responses to a question about qualities that children can be encouraged to learn at home. Respondents are asked which of up to five qualities they consider to be especially important. The exact wording was as follows: "Here is a list of qualities that children can be encouraged to learn at home. Which, if any, do you consider to be especially important? Please choose up to five! (Code five mentions at the maximum): Unselfishness". The respective measure takes value 1 if respondents mention unselfishness, and 0 otherwise.

Order of Questions. The order of survey items was as follows: Patience, prosociality, risk-taking, trust. The questionnaire with all questions and their exact order are publicly available at the website of the World Values Survey (`https://www.worldvaluessurvey.org`).

S.2 Supplementary Appendix: Additional Figures and Tables

Table S.1: List of countries in sample: Global Preferences Survey

Country	In OECD (as of 2012)	Country	In OECD (as of 2012)
Afghanistan	no	Kazakhstan	no
Algeria	no	Kenya	no
Argentina	no	Lithuania	no
Australia	yes	Malawi	no
Austria	yes	Mexico	yes
Bangladesh	no	Morocco	no
Bolivia	no	Netherlands	yes
Bosnia & Herzegovina	no	Nicaragua	no
Botswana	no	Nigeria	no
Brazil	no	Pakistan	no
Cambodia	no	Peru	no
Cameroon	no	Philippines	no
Canada	yes	Poland	yes
Chile	yes	Portugal	yes
China	no	Republic of Korea	yes
Colombia	no	Republic of Moldova	no
Costa Rica	no	Romania	no
Croatia	no	Russian Federation	no
Czechia	yes	Rwanda	no
Egypt	no	Saudi Arabia	no
Estonia	yes	Serbia	no
Finland	yes	South Africa	no
France	yes	Spain	yes
Georgia	no	Sri Lanka	no
Germany	yes	Suriname	no
Ghana	no	Sweden	yes
Greece	yes	Switzerland	yes
Guatemala	no	Thailand	no
Haiti	no	Türkiye	yes
Hungary	yes	Uganda	no
India	no	Ukraine	no
Indonesia	no	United Arab Emirates	no
Iran	no	United Kingdom	yes
Iraq	no	United Republic of Tanzania	no
Israel	yes	United States of America	yes
Italy	yes	Venezuela	no
Japan	yes	Vietnam	no
Jordan	no	Zimbabwe	no

Note: This table lists all 76 countries in the sample and their member status in the Organisation for Economic Co-operation and Development (OECD) as of 2012.

Table S.2: List of countries in sample: World Values Survey

Country	Waves	In OECD (as of 2012)	Country	Waves	In OECD (as of 2012)
Albania	3, 4	no	Malaysia	5–7	no
Algeria	4, 6	no	Maldives	7	no
Andorra	5, 7	no	Mali	5	no
Argentina	1–7	no	Mexico	2–7	yes
Armenia	3, 6, 7	no	Mongolia	7	no
Australia	1, 3, 5–7	yes	Montenegro	3, 4	no
Azerbaijan	3, 6	no	Morocco	4–7	no
Bangladesh	3, 4, 7	no	Myanmar	7	no
Belarus	2, 3, 6	no	Netherlands	5–7	yes
Bolivia	7	no	New Zealand	3, 5–7	yes
Bosnia & Herzegovina	4	no	Nicaragua	7	no
Brazil	3, 5–7	no	Nigeria	2–4, 6, 7	no
Bulgaria	3, 5	no	North Macedonia	3, 4	no
Burkina Faso	5	no	Norway	3, 5	yes
Canada	1, 2, 4, 5, 7	yes	Pakistan	3, 4, 6, 7	no
Chile	2–7	yes	Palestine	6	no
China	2–7	no	Peru	3–7	no
Colombia	3, 5–7	no	Philippines	3, 4, 6, 7	no
Croatia	3	no	Poland	3, 5, 6	yes
Cyprus	5–7	no	Puerto Rico	3, 4, 7	no
Czechia	2, 3, 7	yes	Qatar	6	no
Dominican Republic	3	no	Republic of Korea	2–7	yes
Ecuador	6, 7	no	Republic of Moldova	3–5	no
Egypt	4–7	no	Romania	3, 5–7	no
El Salvador	3	no	Russian Federation	2, 3, 5–7	no
Estonia	3, 6	yes	Rwanda	5, 6	no
Ethiopia	5, 7	no	Saudi Arabia	4	no
Finland	3, 5	yes	Serbia	3–5, 7	no
France	5	yes	Singapore	4, 6, 7	no
Georgia	3, 5, 6	no	Slovakia	2, 3, 7	yes
Germany	3, 5–7	yes	Slovenia	3, 5, 6	yes
Ghana	5, 6	no	South Africa	2–6	no
Greece	7	yes	Spain	2–6	yes
Guatemala	5, 7	no	Sweden	1–6	yes
Hong Kong	5–7	no	Switzerland	2, 3, 5	yes
Hungary	3, 5	yes	Taiwan	3, 5–7	no
India	2–6	no	Tajikistan	7	no
Indonesia	4, 5, 7	no	Thailand	5–7	no
Iran	4, 5, 7	no	Trinidad & Tobago	5, 6	no
Iraq	4–7	no	Tunisia	6, 7	no
Israel	4	yes	Türkiye	2–7	yes
Italy	5	yes	Uganda	4	no
Japan	1–7	yes	Ukraine	3, 5–7	no
Jordan	4–7	no	United Kingdom	3, 5, 7	yes
Kazakhstan	6, 7	no	United Republic of Tanzania	4	no
Kenya	7	no	United States of America	1–7	yes
Kuwait	6	no	Uruguay	3, 5–7	no
Kyrgyzstan	4, 6, 7	no	Uzbekistan	6	no
Latvia	3	no	Venezuela	3, 4, 7	no
Lebanon	6, 7	no	Vietnam	4, 5, 7	no
Libya	6, 7	no	Yemen	6	no
Lithuania	3	no	Zambia	5	no
Macao	7	no	Zimbabwe	4, 6, 7	no

Note: This table lists all 106 countries and territories in the sample, the waves in which they were observed, and their member status in the Organisation for Economic Co-operation and Development (OECD) as of 2012.

Table S.3: Descriptive statistics

Variable	Observations	Mean	Standard deviation	Minimum	Maximum
**Panel (a): Gender preference gaps in Global Preferences Survey**					
Patience	513	0.19	0.16	0.00	0.99
Patience (age-corrected)	513	0.12	0.12	0.00	0.78
Risk-taking	513	0.26	0.18	0.00	1.01
Risk-taking (age-corrected)	513	0.12	0.13	0.00	0.92
Trust	512	0.19	0.17	0.00	1.04
Trust (age-corrected)	512	0.13	0.13	0.00	1.19
Prosociality	513	0.12	0.11	0.00	0.86
Prosociality (age-corrected)	513	0.09	0.08	0.00	0.54
**Panel (b): Gender preference gaps in World Values Survey**					
Patience	1,792	0.14	0.13	0.00	1.42
Patience (age-corrected)	1,792	0.09	0.09	0.00	0.92
Risk-taking	720	0.25	0.17	0.00	1.24
Risk-taking (age-corrected)	720	0.09	0.11	0.00	1.18
Trust	1,791	0.13	0.13	0.00	1.28
Trust (age-corrected)	1,791	0.09	0.09	0.00	0.76
Prosociality	1,780	0.13	0.13	0.00	1.32
Prosociality (age-corrected)	1,780	0.09	0.09	0.00	1.48

Note: This table shows descriptive statistics for gender preference gaps based on 513 gender gaps covering 76 countries and observed over up to 7 birth decades in the Global Preferences Survey (Panel a) and based on 1,792 gender gaps covering 106 countries and territories observed over up to 7 waves and 7 birth decades in the World Values Survey (Panel b). Each gender gap corresponds to a unique country-period-cohort cell. Reported numbers reflect the absolute difference in means between men and women within birth decades, where means are calculated for each gender within country-, period-, birth decade-specific cells. Data in the Global Preferences Survey and the World Values Survey are standardized to mean 0 and standard deviation 1 on the respective global samples, and gender preference gaps are expressed in standard deviations of the corresponding preference measures.

Table S.4: Descriptive statistics by OECD member status

	OECD countries			Non-OECD countries		
Variable	Observations	Mean	Standard deviation	Observations	Mean	Standard deviation
**Panel (a): Gender preference gaps in Global Preferences Survey**						
Patience	174	0.23	0.20	339	0.16	0.14
Patience (age-corrected)	174	0.14	0.13	339	0.11	0.11
Risk-taking	174	0.29	0.17	339	0.25	0.19
Risk-taking (age-corrected)	174	0.11	0.11	339	0.13	0.14
Trust	174	0.18	0.16	338	0.19	0.18
Trust (age-corrected)	174	0.11	0.09	338	0.14	0.15
Prosociality	174	0.12	0.10	339	0.12	0.11
Prosociality (age-corrected)	174	0.08	0.07	339	0.09	0.08
**Panel (b): Gender preference gaps in World Values Survey**						
Patience	590	0.14	0.12	1,202	0.13	0.13
Patience (age-corrected)	590	0.09	0.09	1,202	0.09	0.09
Risk-taking	227	0.26	0.14	493	0.25	0.18
Risk-taking (age-corrected)	227	0.08	0.07	493	0.10	0.12
Trust	595	0.14	0.12	1,196	0.13	0.13
Trust (age-corrected)	595	0.09	0.08	1,196	0.09	0.09
Prosociality	584	0.13	0.13	1,196	0.13	0.13
Prosociality (age-corrected)	584	0.09	0.10	1,196	0.09	0.09

Note: This table shows descriptive statistics for gender preference gaps by Organisation for Economic Co-operation and Development (OECD) member status. The descriptive statistics are based on 513 gender gaps covering 76 countries and observed over 7 birth decades in the Global Preferences Survey (Panel a) and based on 1,792 gender gaps covering 106 countries and territories observed over 7 waves and 7 birth decades in the World Values Survey (Panel b). Each gender gap corresponds to a unique country-period-cohort cell. Reported numbers reflect the absolute difference in means between men and women within birth decades, where means are calculated for each gender within country-, period-, birth decade-specific cells. Data in the Global Preferences Survey and the World Values Survey are standardized to mean 0 and standard deviation 1 on the respective global samples, and gender preference gaps are expressed in standard deviations of the corresponding preference measures.

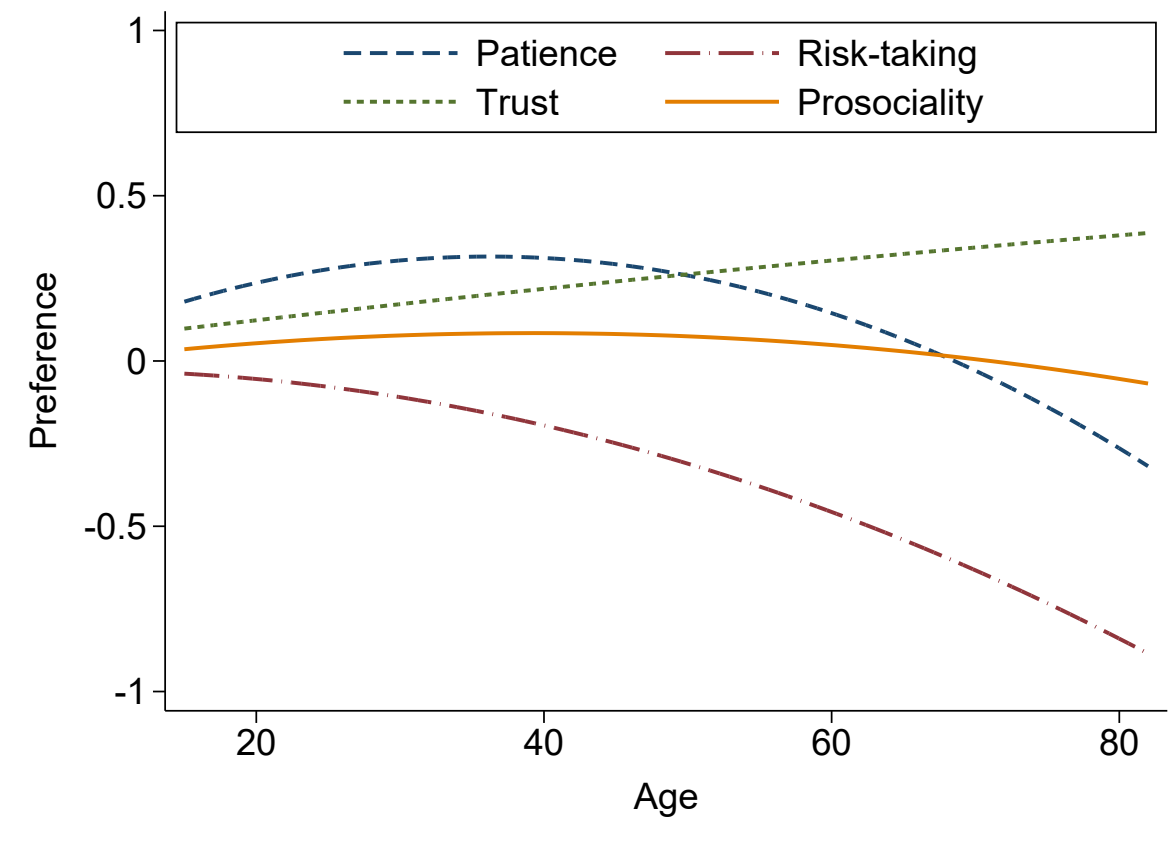

(a) OECD countries: Men

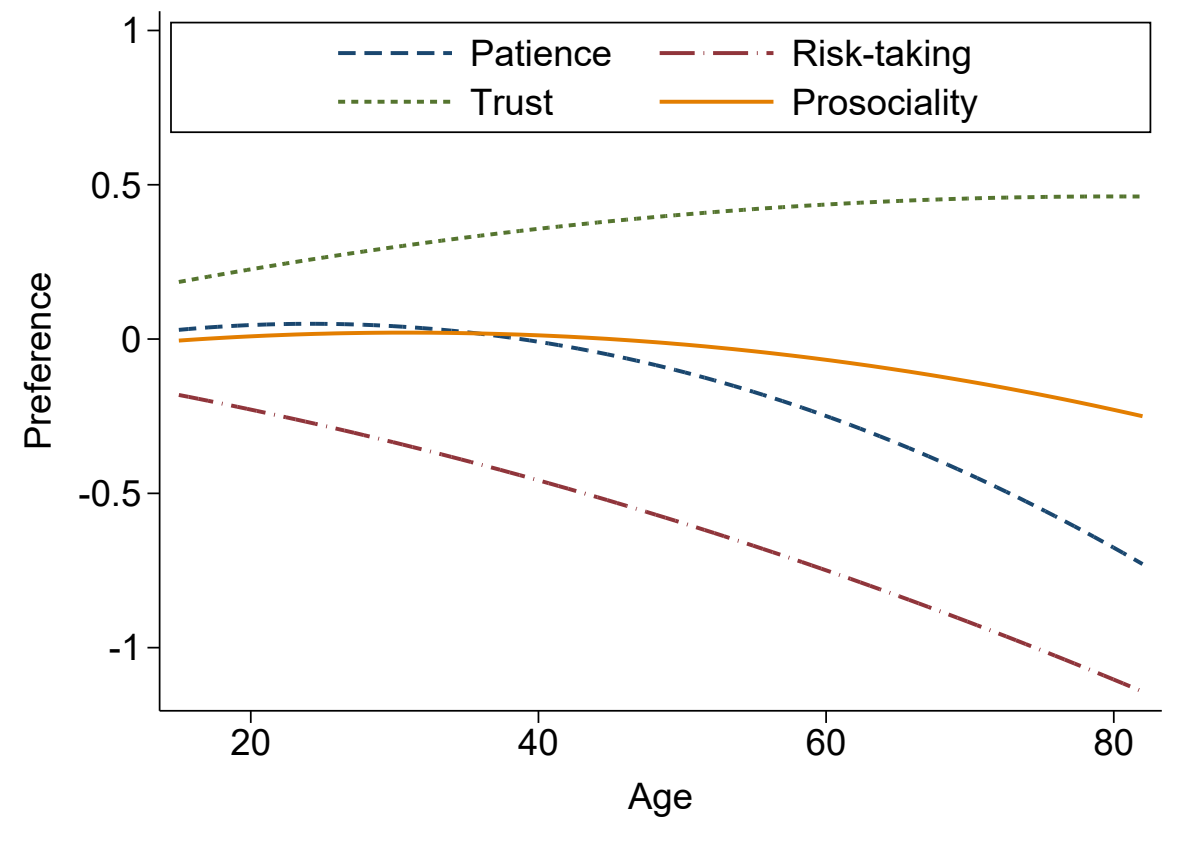

(b) OECD countries: Women

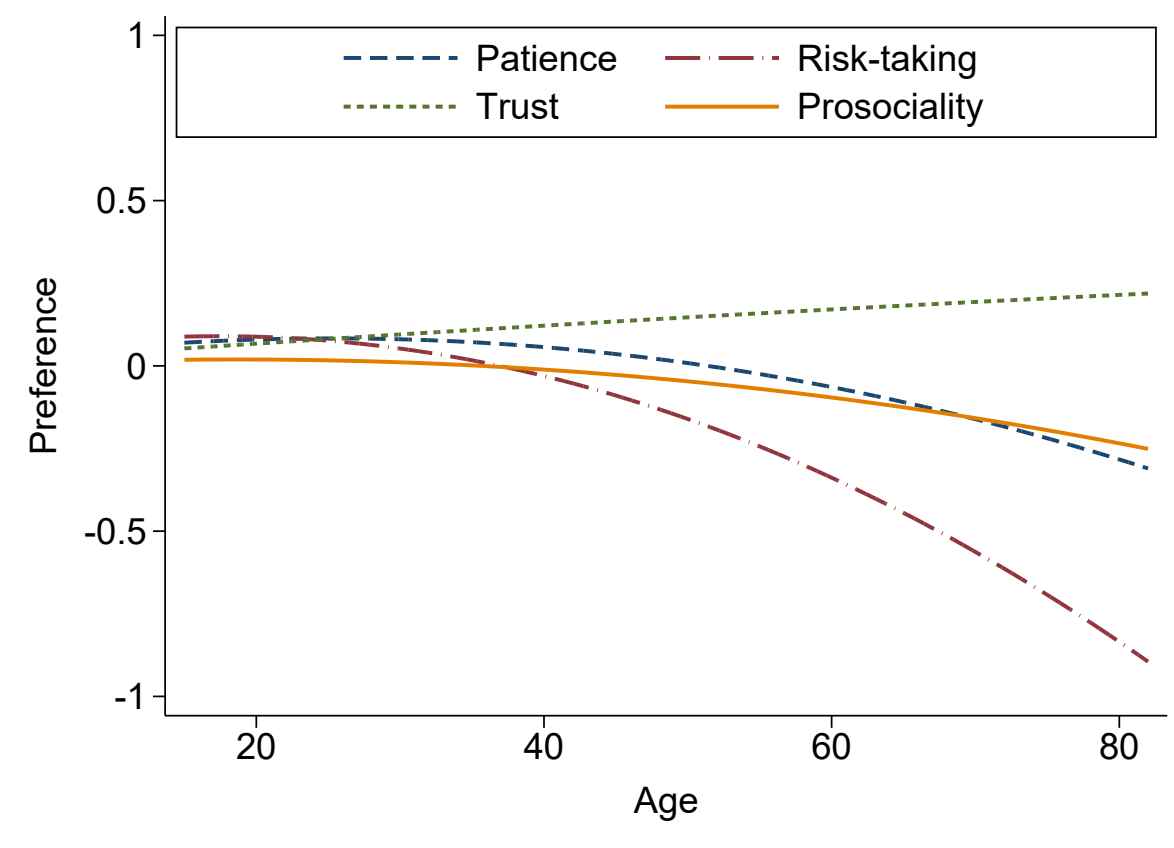

(c) Non-OECD countries: Men

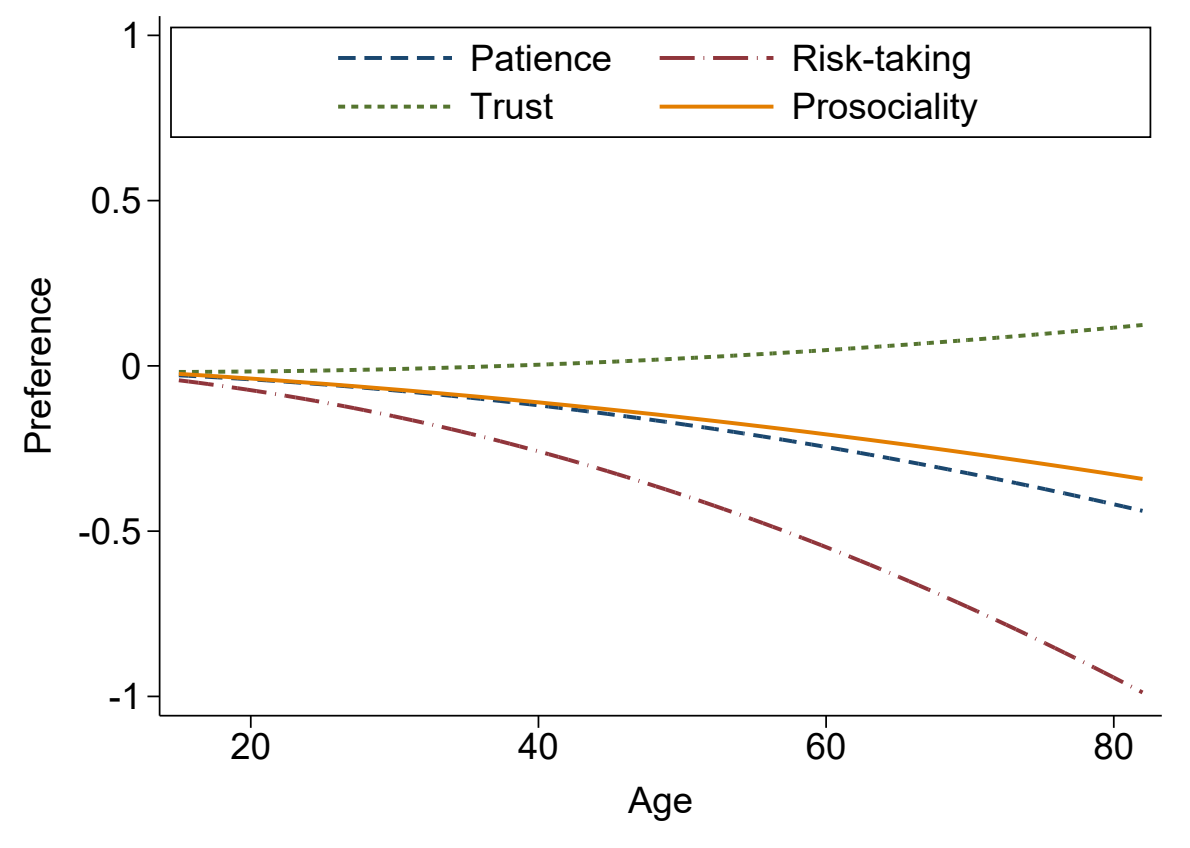

(d) Non-OECD countries: Women

Note: This figure shows average age profiles in individual preferences by gender and OECD member status in the Global Preferences Survey. Panel (a) shows men in OECD countries, Panel (b) shows women in OECD countries, Panel (c) shows men in non-OECD countries, and Panel (d) shows women in non-OECD countries. Each graph is an augmented-component-plus-residuals plot showing how preferences vary with age. The vertical axis shows the components of preferences predicted by a quadratic age trend and the residuals, which were both derived from an individual-level regression of the respective preference measures on a quadratic age trend and country fixed effects. All preference measures are standardized to mean 0 and standard deviation 1 on the respective global samples such that age profiles are expressed in standard deviations of the corresponding preference measures.

Figure S.1: Age profiles by economic development and gender: Global Preferences Survey

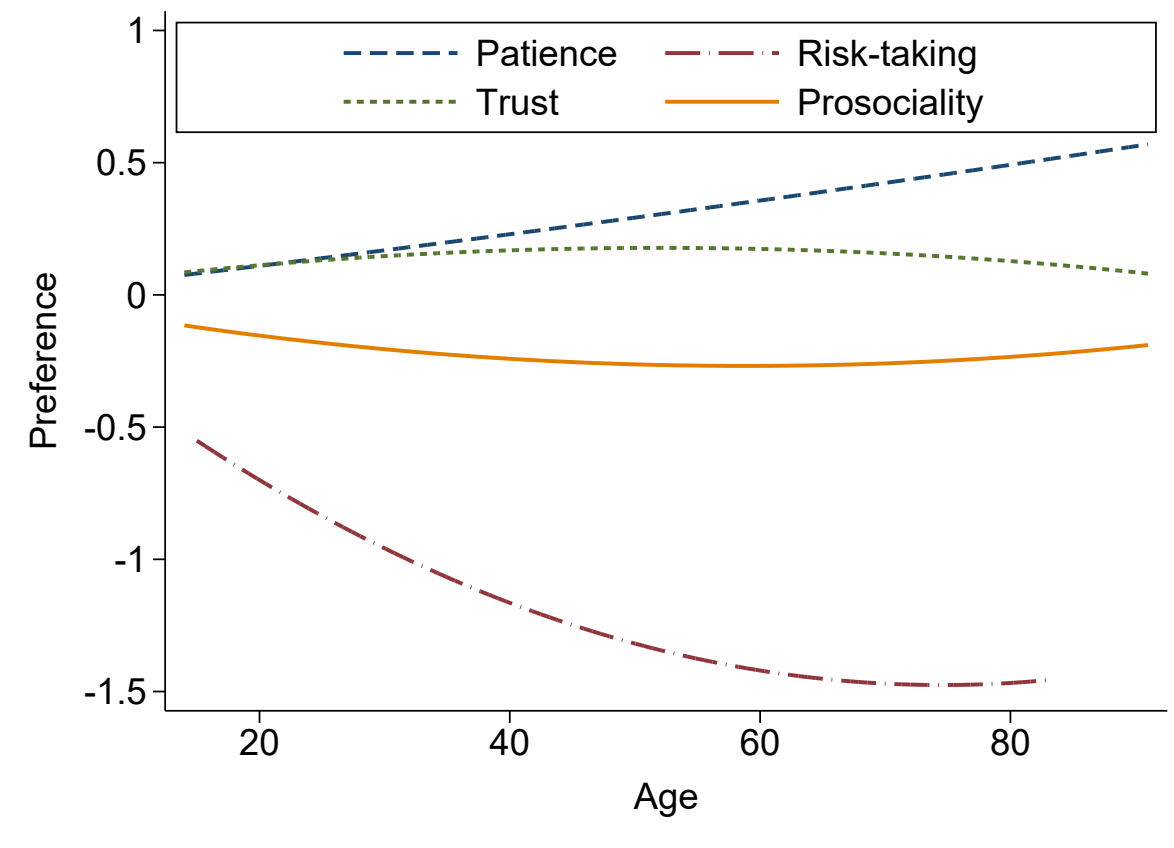

(a) OECD countries: Men

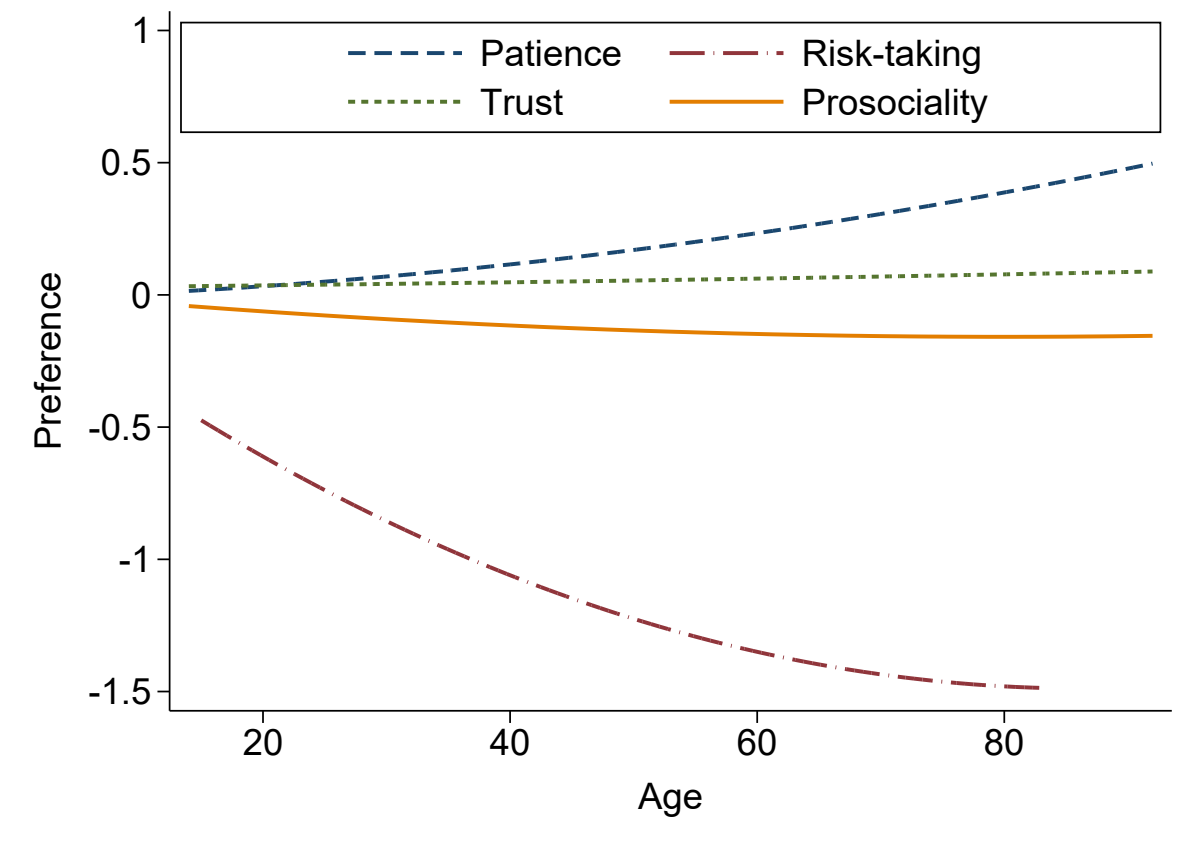

(b) OECD countries: Women

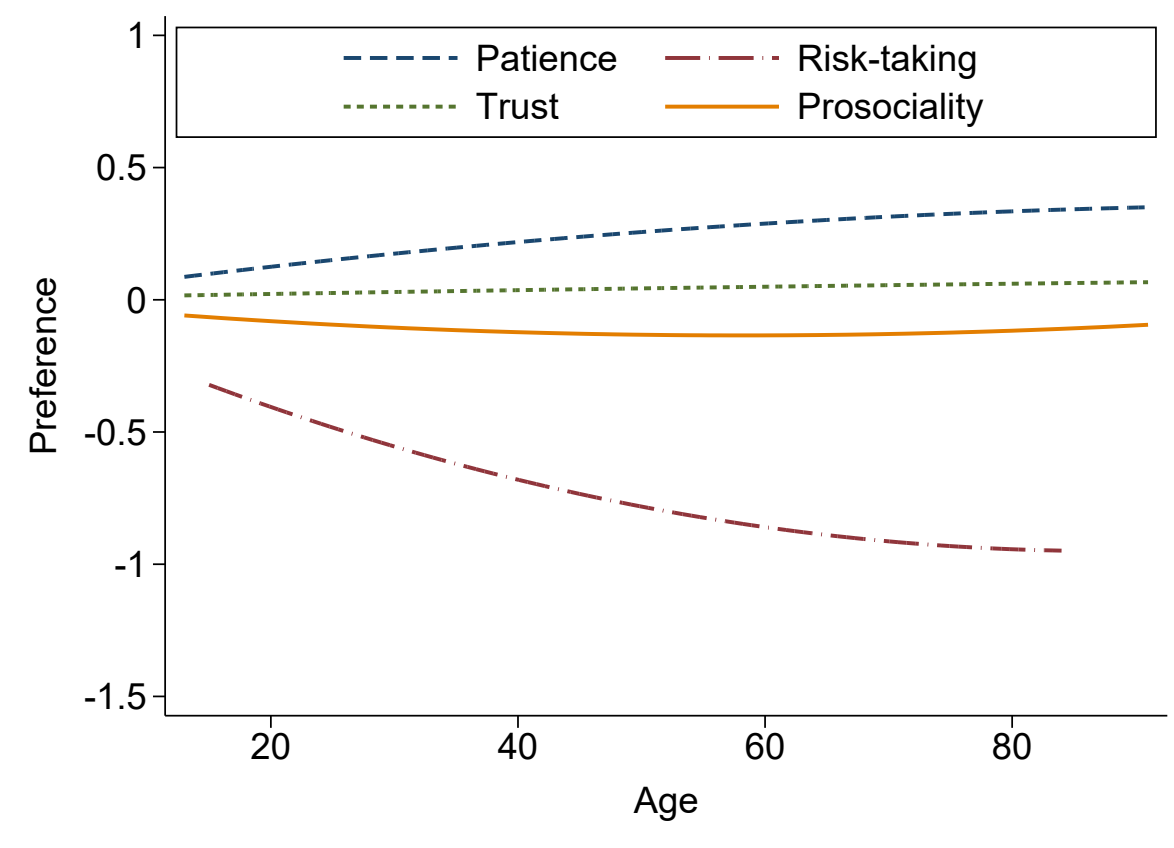

(c) Non-OECD countries: Men

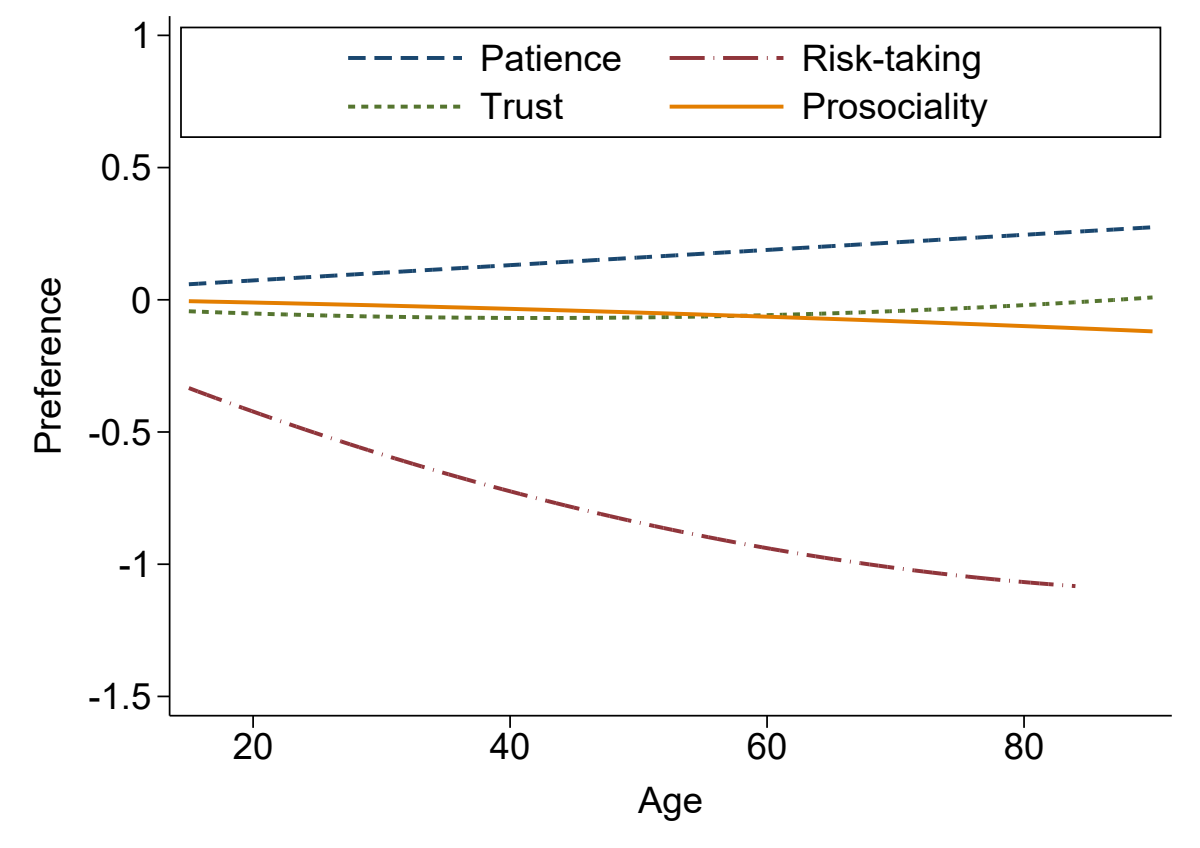

(d) Non-OECD countries: Women

Note: This figure shows average age profiles in individual preferences by gender and OECD member status in the World Values Survey. Panel (a) shows men in OECD countries, Panel (b) shows women in OECD countries, Panel (c) shows men in non-OECD countries, and Panel (d) shows women in non-OECD countries. Each graph is an augmented component plus residuals plot showing how preferences vary with age. The vertical axis shows the components of preferences predicted by a quadratic age trend and the residuals, which were both derived from an individual-level regression of the respective preference measures on a quadratic age trend and country-period fixed effects. All preference measures are standardized to mean 0 and standard deviation 1 on the respective global samples, such that age profiles are expressed in standard deviations of the corresponding preference measures.

Figure S.2: Age profiles by economic development and gender: World Values Survey

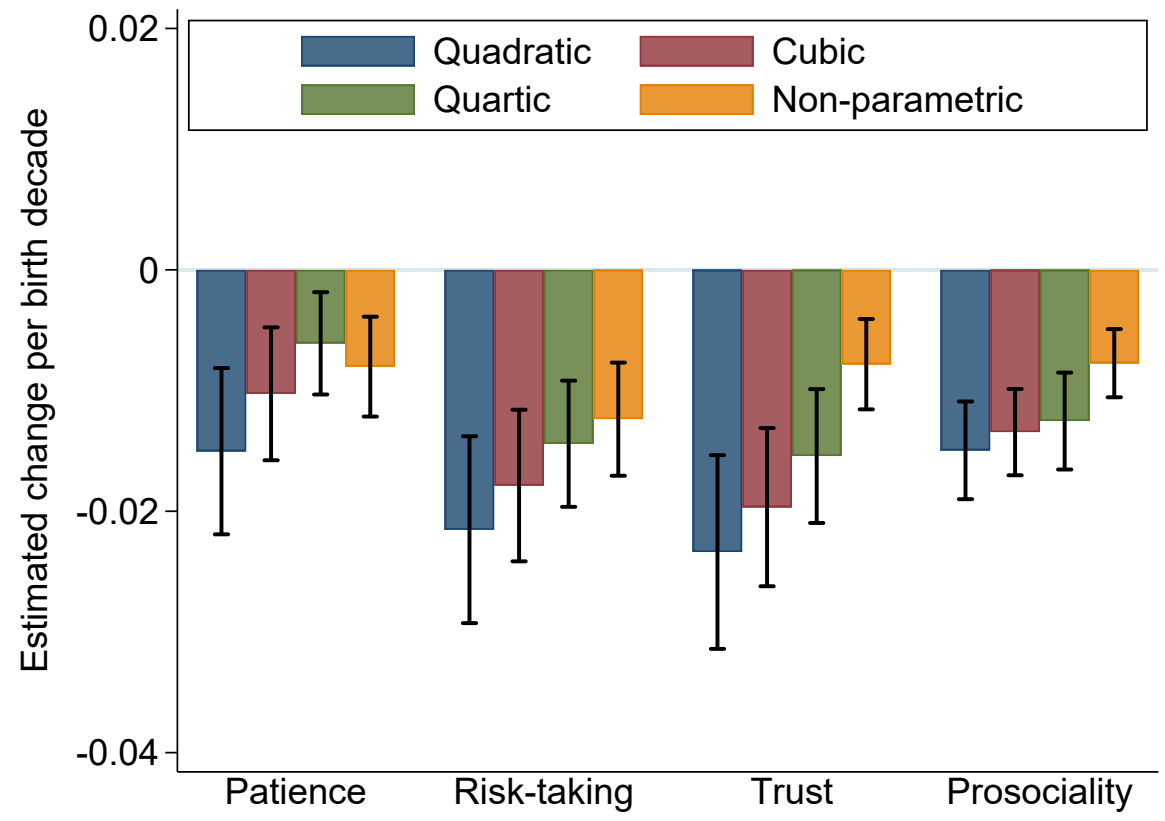

(a) Global Preferences Survey

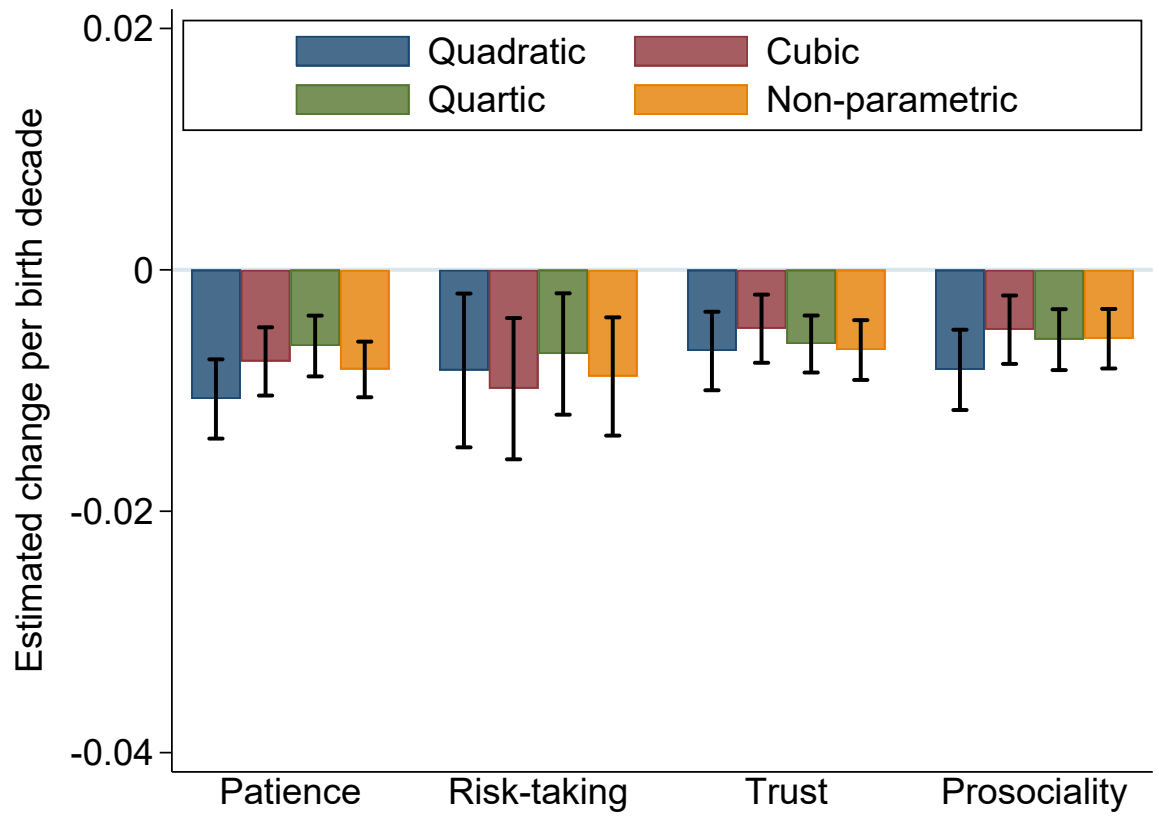

(b) World Values Survey

Note: This figure shows coefficient estimates and 95% confidence intervals from regressions of within-country gender preference gaps on linear birth decade trends in the Global Preferences Survey (Panel a) and in the World Values Survey (Panel b). The coefficients reflect the estimated changes in gender preference gaps per birth decade (i.e., cohort trends). An observation is a within-country gender gap per birth decade. Data for the Global Preferences Survey (GPS) comprise 76 countries observed over 7 birth decades. Data for the World Values Survey (WVS) comprise 106 countries observed over 7 birth decades and up to 7 survey waves (1981–2022). The dependent variable is the absolute difference in age-corrected preference means between men and women, where means are separately calculated for each gender within country-, period-, birth decade-specific cells. The age correction in Panel (a) is performed with country-specific age trends that are (i) quadratic, (ii) cubic, (iii) quartic, or (iv) non-parametric (derived from estimates based on 10-year age bins). The age correction in Panel (b) is performed with country wave-specific age trends that are (i) quadratic, (ii) cubic, (iii) quartic, or (iv) non-parametric (derived from estimates based on 10-year age bins). All preference measures are standardized to mean 0 and standard deviation 1 on the respective global samples such that gender preference gaps are expressed in standard deviations of the corresponding preference measures (see Methods). Specifications in Panel (a) include country fixed effects, whereas specifications in Panel (b) include country-period fixed effects. Confidence intervals are based on standard errors that are clustered at the country level.

Figure S.3: Estimated changes in gender gaps per birth decade: Alternative age corrections

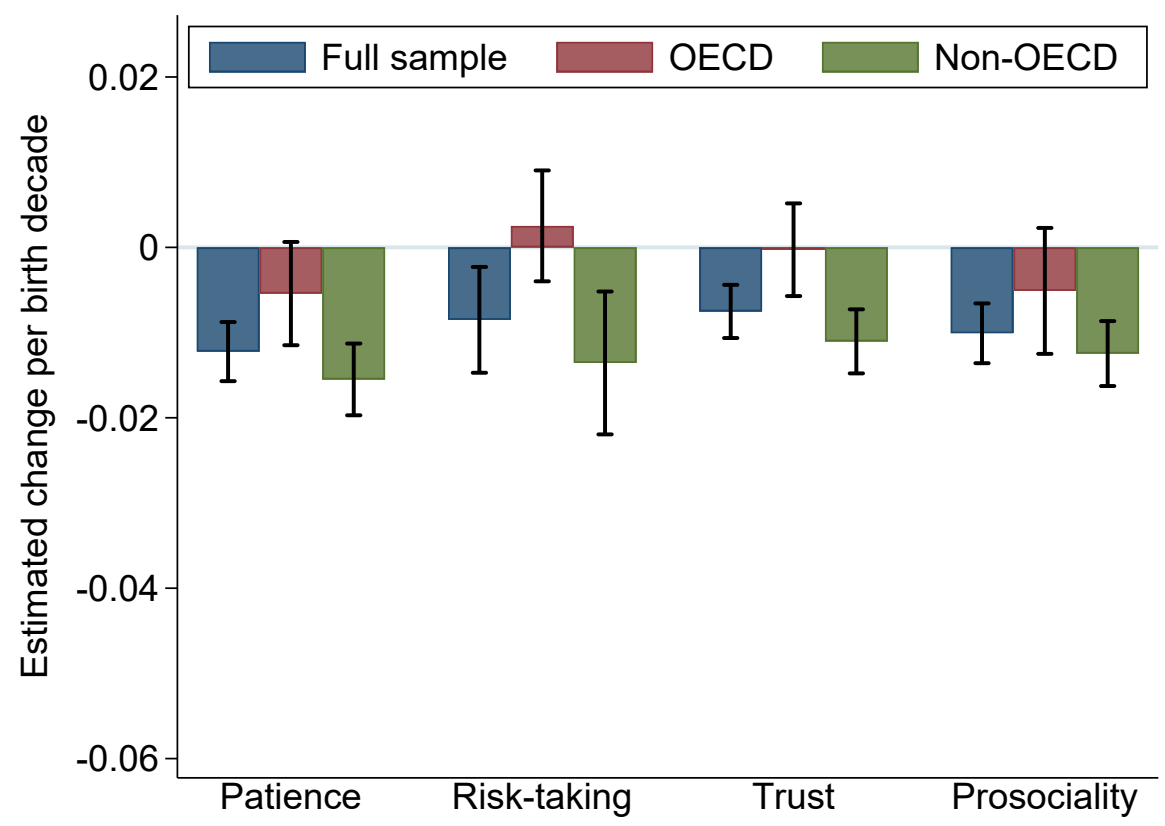

(a) By economic development

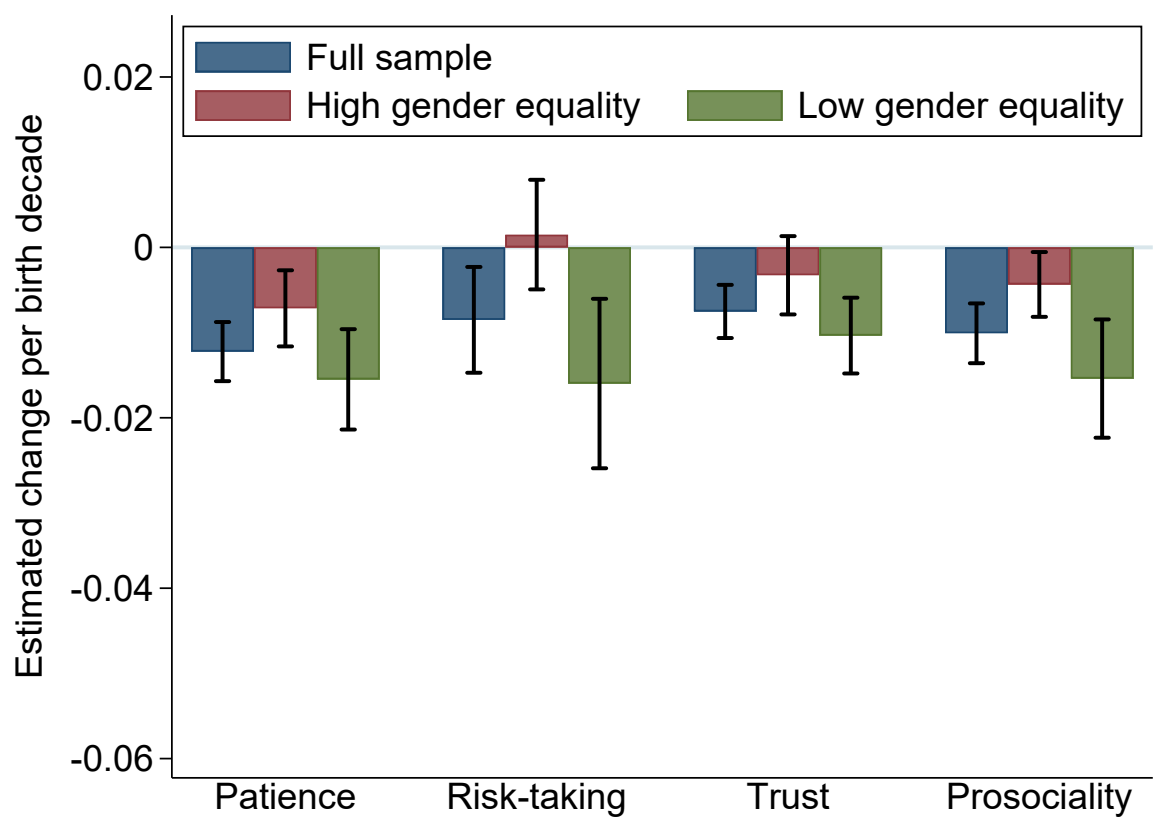

(b) By gender equality

Note: This figure shows coefficient estimates and 95% confidence intervals from regressions of within-country gender preference gaps on linear birth decade trends in the World Values Survey. The coefficients reflect the estimated changes in gender preference gaps per birth decade (i.e., cohort trends). The data comprise 106 countries observed over 7 birth decades and up to 7 survey waves (1981–2022). An observation is a within-country gender gap per birth decade. The dependent variable is the absolute difference in means of age-corrected preferences between men and women, where means are separately calculated for each gender within country-, period-, birth decade-specific cells. Age-corrected preference measures have been purged of country-, period-, gender-specific age profiles. All preference measures are standardized to mean 0 and standard deviation 1 on the respective global samples such that gender preference gaps are expressed in standard deviations of the corresponding preference measures (see Methods). The figure shows heterogeneity in the estimated changes in gender preference gaps per birth decade by level of economic development in terms of OECD member status (Panel a) and by level of gender equality (Panel b). A country is defined as gender-equal if its gender parity score in the World Economic Forum's gender parity index (**?**) exceeds the median. All specifications include country-period fixed effects. Confidence intervals are based on standard errors that are clustered at the country level.

Figure S.4: Estimated changes in gender gaps per birth decade by economic development and gender equality

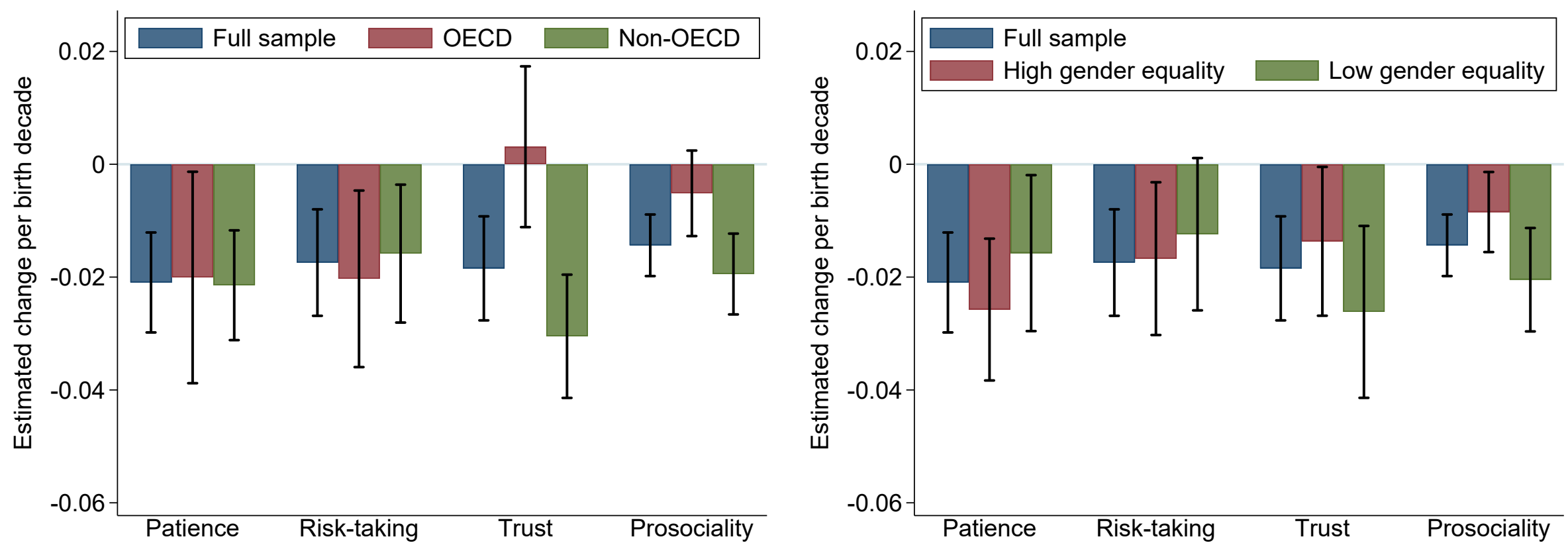

(a) By economic development (b) By gender equality

Note: This figure shows coefficient estimates and 95% confidence intervals from regressions of within-country gender preference gaps on linear birth decade trends in the Global Preferences Survey. The coefficients reflect the estimated changes in gender preference gaps per birth decade (i.e., cohort trends). The data comprise 76 countries observed over 7 birth decades. An observation is a within-country gender gap per birth decade. The dependent variable is the absolute difference in means of preferences between men and women, where means are separately calculated for each gender within country-, period-, birth decade-specific cells. All preference measures are standardized to mean 0 and standard deviation 1 on the respective global samples such that gender preference gaps are expressed in standard deviations of the corresponding preference measures (see Methods). The figure shows heterogeneity in the estimated changes in gender preference gaps per birth decade by level of economic development in terms of OECD member status (Panel a) and by level of gender equality (Panel b). A country is defined as gender-equal if its gender parity score in the World Economic Forum's gender parity index (**?**) exceeds the median. All specifications include country fixed effects. Confidence intervals are based on standard errors that are clustered at the country level.

Figure S.5: Estimated changes in gender gaps per birth decade by economic development and gender equality: Results without correction for age profiles in preference measures

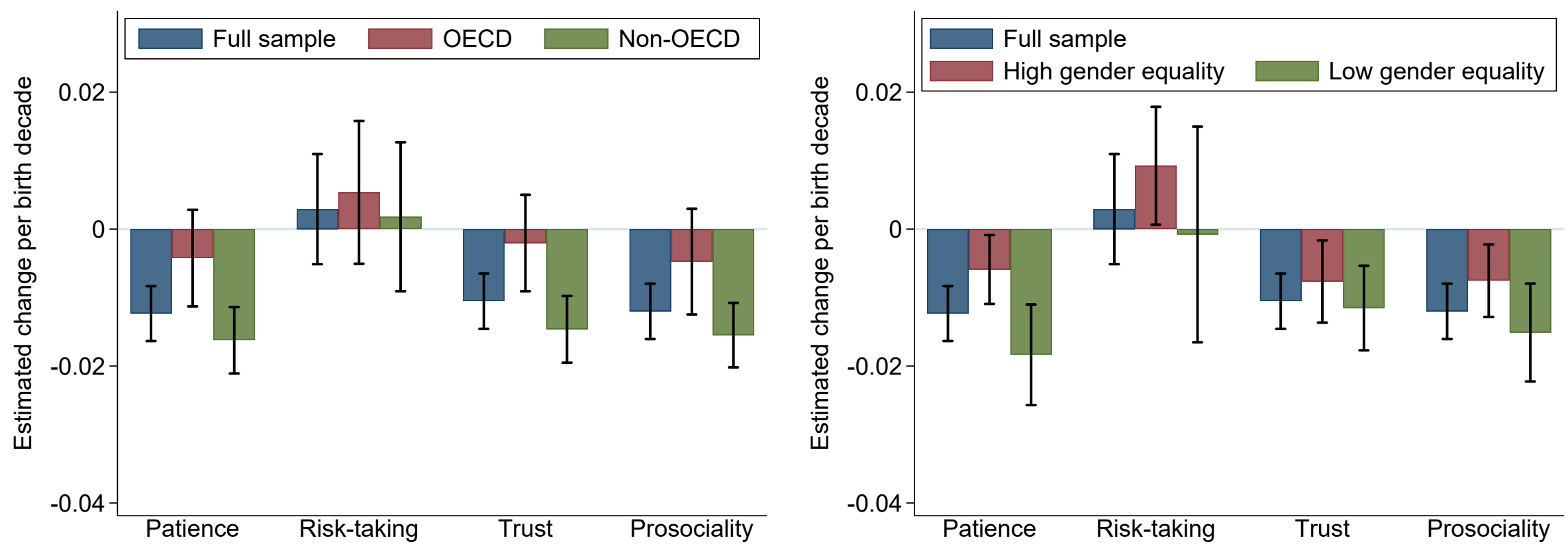

(a) By economic development (b) By gender equality

Note: This figure shows coefficient estimates and 95% confidence intervals from regressions of within-country gender preference gaps on linear birth decade trends in the World Values Survey. The coefficients reflect the estimated changes in gender preference gaps per birth decade (i.e., cohort trends). The data comprise 106 countries observed over 7 birth decades and up to 7 survey waves (1981–2022). An observation is a within-country gender gap per birth decade. The dependent variable is the absolute difference in means of preferences between men and women, where means are separately calculated for each gender within country-, period-, birth decade-specific cells. All preference measures are standardized to mean 0 and standard deviation 1 on the respective global samples such that gender preference gaps are expressed in standard deviations of the corresponding preference measures (see Methods). The figure shows heterogeneity in the estimated changes in gender preference gaps per birth decade by level of economic development in terms of OECD member status (Panel a) and by level of gender equality (Panel b). A country is defined as gender-equal if its gender parity score in the World Economic Forum's gender parity index (**?**) exceeds the median. All specifications include country-period fixed effects. Confidence intervals are based on standard errors that are clustered at the country level.

Figure S.6: Estimated changes in gender gaps per birth decade by economic development and gender equality: Results without correction for age profiles in preference measures

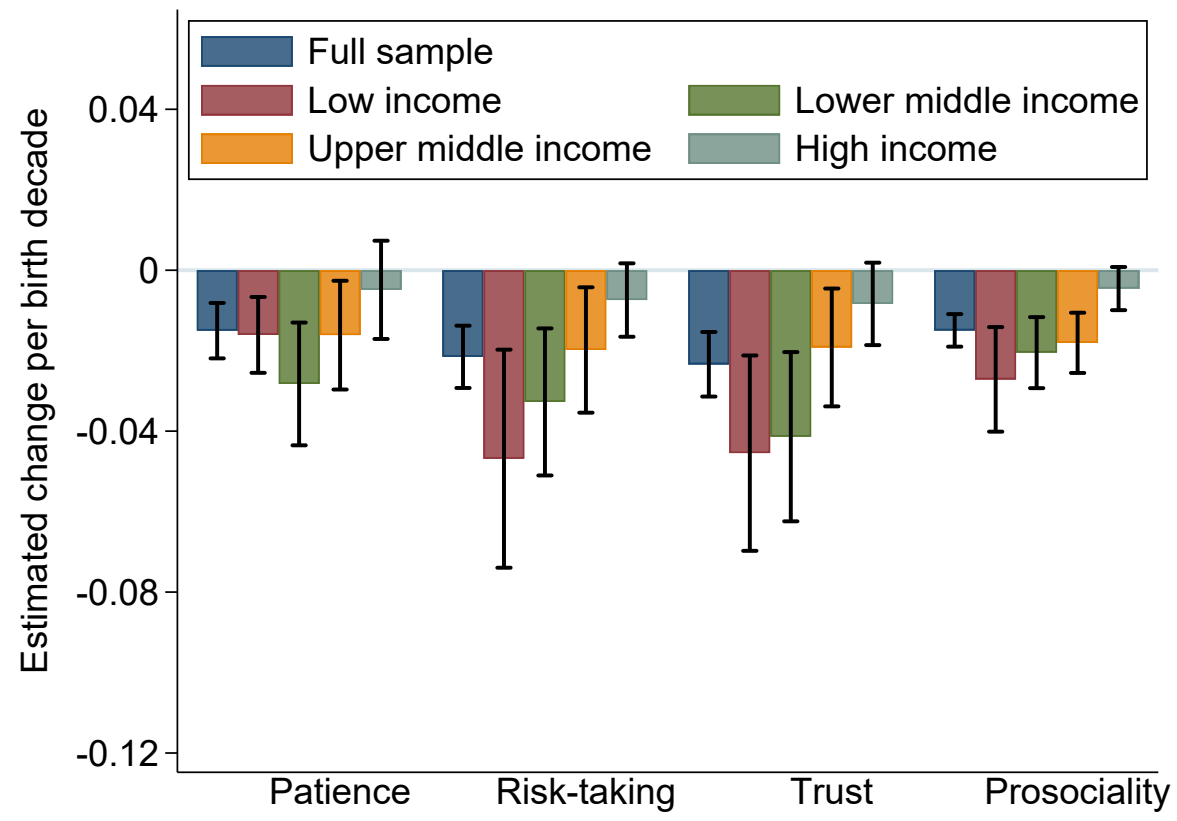

(a) Global Preferences Survey

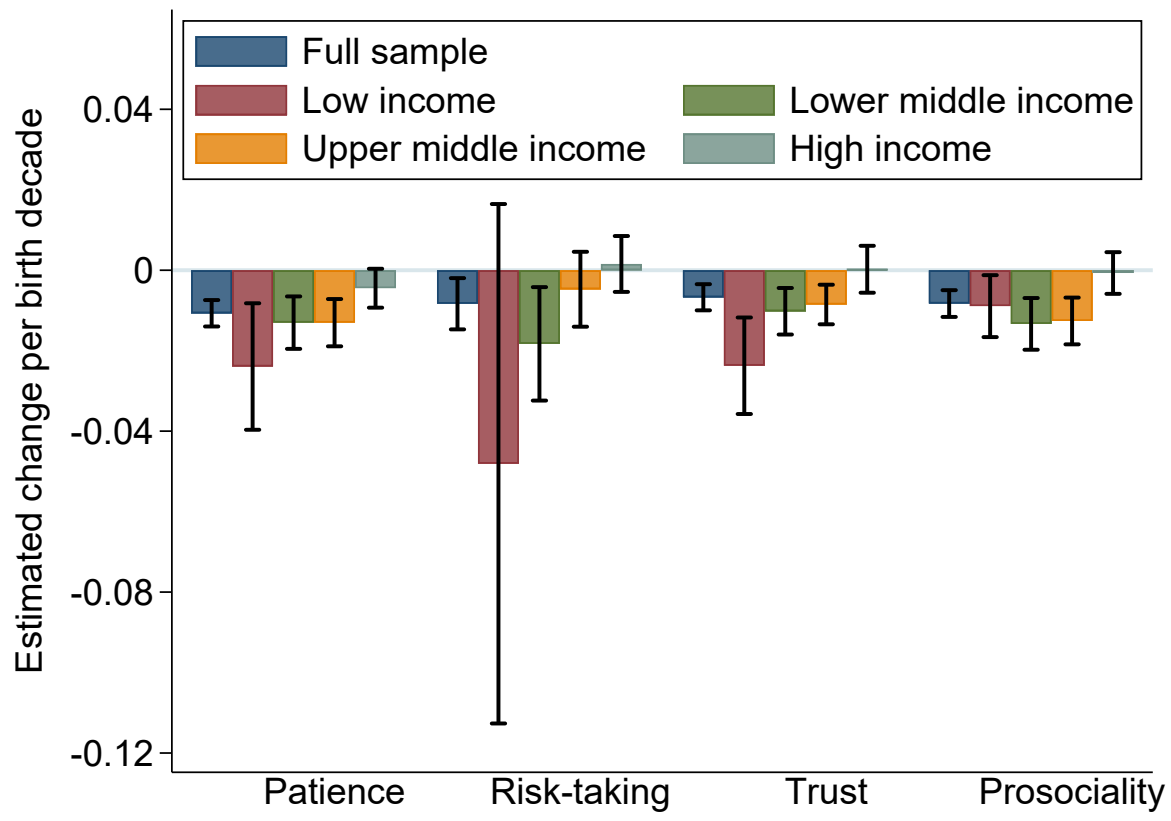

(b) World Values Survey

Note: This figure shows coefficient estimates and 95% confidence intervals from regressions of within-country gender preference gaps on linear birth decade trends by economic development. Countries are split into income groups following the World Bank's income classification for 2012. The coefficients reflect the estimated changes in gender preference gaps per birth decade (i.e., cohort trends). An observation is a within-country gender gap per birth decade. Data for the Global Preferences Survey (Panel a) comprise 76 countries observed over 7 birth decades. Data for the World Values Survey (Panel b) comprise 106 countries observed over 7 birth decades and up to 7 survey waves during 1981–2022. The dependent variable is the absolute difference in means of age-corrected preferences between men and women. Age-corrected preference measures have been purged of country-, gender-specific age profiles in the cross-sectional data from the Global Preferences Survey and of country-, period-, gender-specific age profiles in the longitudinal data from the World Values Survey. All preference measures are standardized to mean 0 and standard deviation 1 on the respective global samples such that gender preference gaps are expressed in standard deviations of the corresponding preference measures (see Methods). Specifications for the Global Preferences Survey include country fixed effects, whereas specifications for the World Values Survey include country-period fixed effects. Confidence intervals are based on standard errors that are clustered at the country level.

Figure S.7: Estimated changes in gender gaps per birth decade by income level

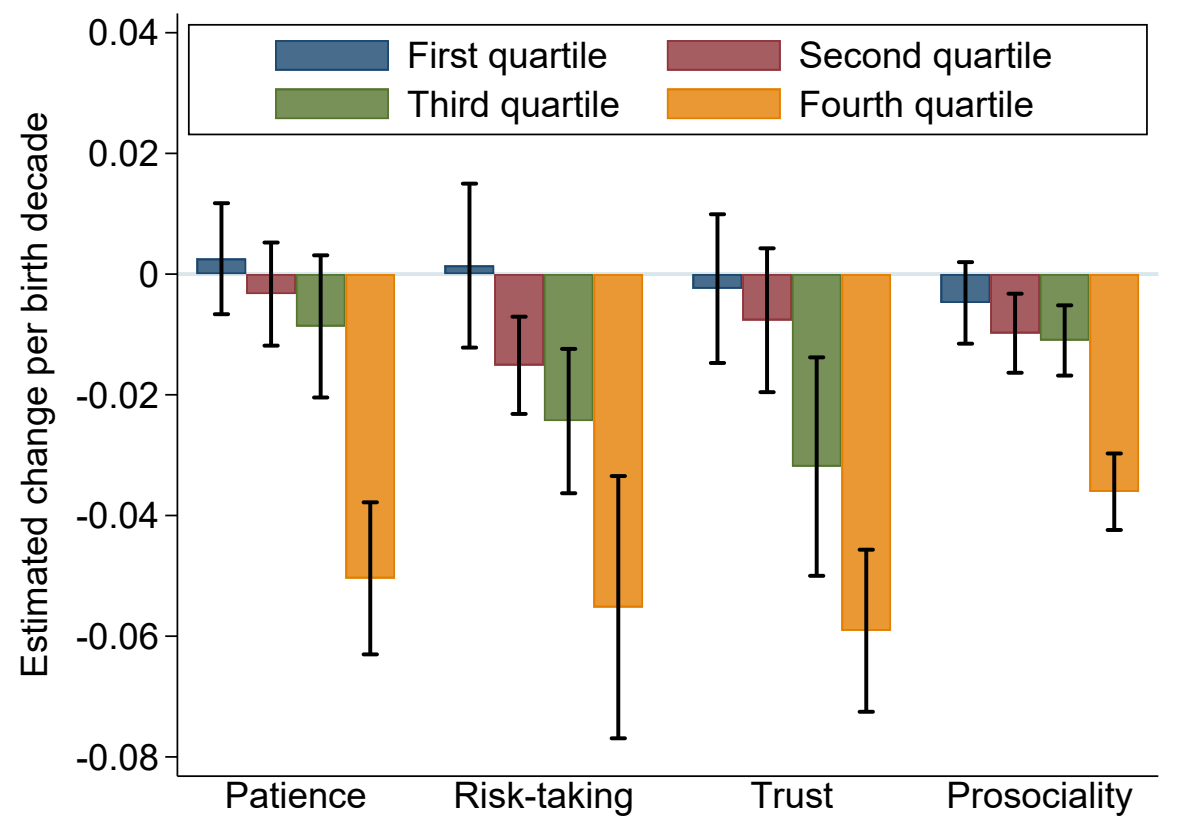

(a) Global Preferences Survey

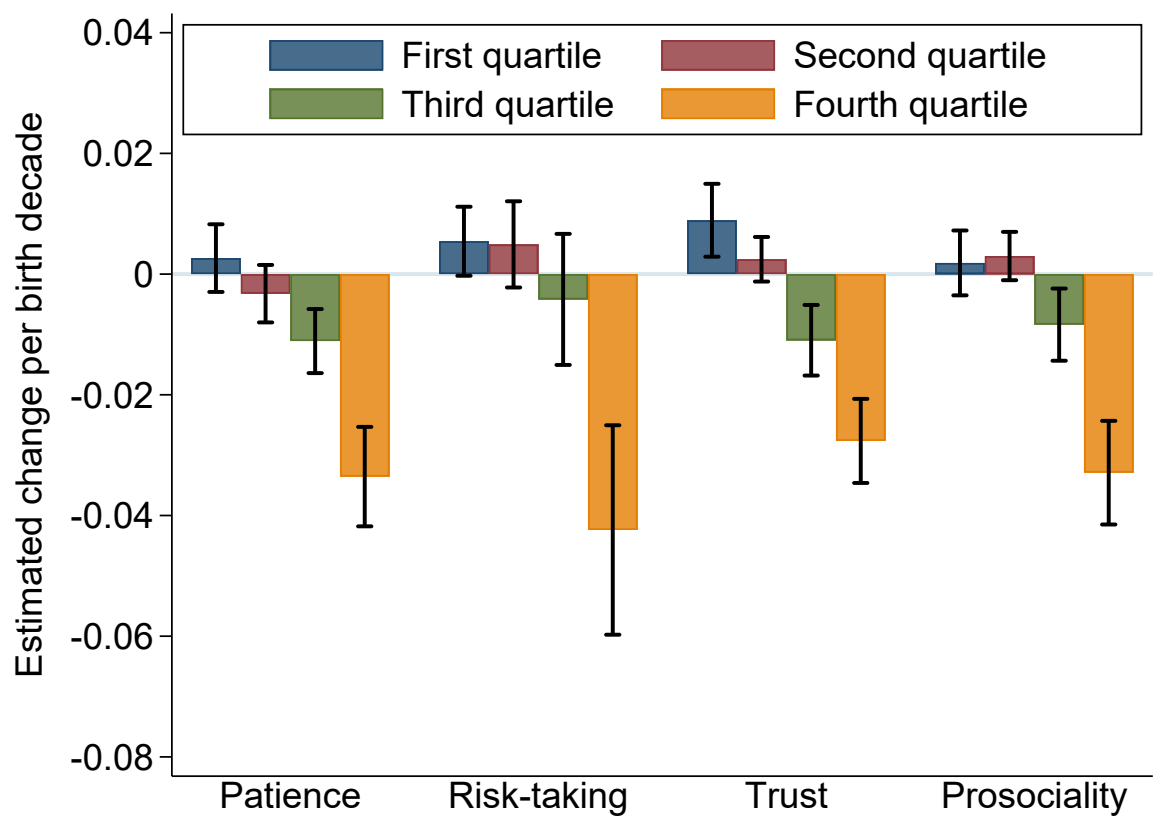

(b) World Values Survey

Note: This figure shows coefficient estimates and 95% confidence intervals from regressions of within-country gender preference gaps on linear birth decade trends by initial gender gaps. Country pairs are grouped into quartiles according to the size of their initial gender gaps, as measured in 1940. An observation is a within-country gender gap per birth decade. Data for the Global Preferences Survey (Panel a) comprise 76 countries observed over 7 birth decades. Data for the World Values Survey (Panel b) comprise 106 countries observed over 7 birth decades and up to 7 survey waves during 1981–2022. The dependent variable is the absolute difference in means of age-corrected preferences between men and women. Age-corrected preference measures have been purged of country-, gender-specific age profiles in the cross-sectional data from the Global Preferences Survey and of country-, period-, gender-specific age profiles in the longitudinal data from the World Values Survey. All preference measures are standardized to mean 0 and standard deviation 1 on the respective global samples such that gender preference gaps are expressed in standard deviations of the corresponding preference measures (see Methods). Specifications for the Global Preferences Survey include country fixed effects, whereas specifications for the World Values Survey include country-period fixed effects. Confidence intervals are based on standard errors that are clustered at the country level.

Figure S.8: Estimated changes in gender gaps per birth decade by initial gender gaps

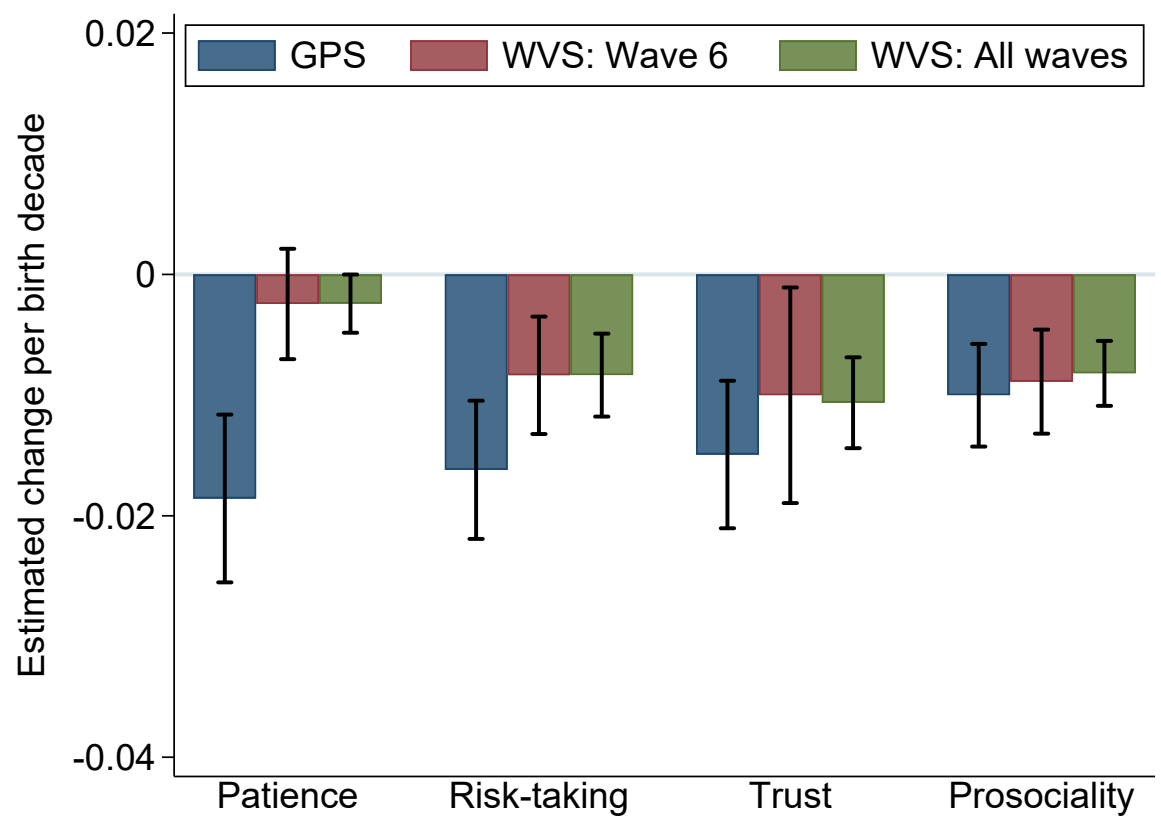

Note: This figure shows coefficient estimates and 95% confidence intervals from regressions of within-country gender preference gaps on linear birth decade trends. The coefficients reflect the estimated changes in gender preference gaps per birth decade (i.e., cohort trends). An observation is a within-country gender gap per birth decade. Data for the Global Preferences Survey (GPS) comprise 76 countries observed over 7 birth decades. Data for the World Values Survey (WVS) comprise 106 countries observed over 7 birth decades and up to 7 survey waves during 1981–2022. All preference measures are standardized to mean 0 and standard deviation 1 on the respective global samples. The dependent variable is the absolute difference in standard deviations of age-corrected preferences between men and women, where standard deviations are separately calculated for each gender within country-, period-, birth decade-specific cells. Age-corrected preference measures have been purged of country-, gender-specific age profiles in the cross-sectional data from the Global Preferences Survey and of country-, period-, gender-specific age profiles in the longitudinal data from the World Values Survey. Specifications for the Global Preferences Survey include country fixed effects, whereas specifications for the World Values Survey include country-period fixed effects. Confidence intervals are based on standard errors that are clustered at the country level.

Figure S.9: Estimated changes in gender variability gaps per birth decade: Global Preferences Survey vs. World Values Survey

Table S.5: Estimated changes in gender gaps per birth decade: Only countries with two or more waves

Gender gap in	Patience (1)	Risk-taking (2)	Trust (3)	Prosociality (4)
Dependent variable: Gender gaps without correction for age profiles in preferences				
Birth decade	-0.012***	0.005	-0.010***	-0.012***
	(0.003)	(0.005)	(0.002)	(0.002)
R^2	0.23	0.30	0.23	0.22
Clusters	77	68	77	77
Observations	1,622	664	1,621	1,610
Dependent variable: Gender gaps with correction for age profiles in preferences				
Birth decade	-0.012***	-0.008**	-0.007***	-0.010***
	(0.002)	(0.003)	(0.002)	(0.002)
R^2	0.27	0.25	0.27	0.27
Clusters	77	68	77	77
Observations	1,622	664	1,621	1,610

Note: This table shows coefficient estimates from ordinary least squares regressions of within-country gender preference gaps on linear birth decade trends. The coefficients reflect the estimated changes in gender preference gaps per birth decade (i.e., cohort trends). Results are shown for 77 countries and territories observed over 7 birth decades in at least 2 (and up to 7) survey waves during 1981–2022 in the World Values Survey. An observation is a within-country gender gap per birth decade. The dependent variable is the absolute difference in means of preferences between men and women (top of panel) or the absolute difference in means of age-corrected preferences between men and women (bottom of panel), where means are separately calculated for each gender within country-, period-, birth decade-specific cells. Age-corrected preference measures have been purged of country-, period-, gender-specific age profiles. All preference measures are standardized to mean 0 and standard deviation 1 on the respective global samples such that gender preference gaps are expressed in standard deviations of the corresponding preference measures (see Methods). All specifications include country-period fixed effects. Standard errors are clustered at the country level and reported in parentheses. Asterisks indicate significance levels: * $p < 0.1$; ** $p < 0.05$; *** $p < 0.01$.

Table S.6: Estimated changes in gender gaps per birth decade: No weighting during age correction and data aggregation

Gender gap in	Patience (1)	Risk-taking (2)	Trust (3)	Prosociality (4)
**Panel (a): Global Preferences Survey**				
Dependent variable: Gender gaps without correction for age profiles in preferences				
Birth decade	-0.018***	-0.016***	-0.018***	-0.017***
	(0.004)	(0.005)	(0.004)	(0.003)
R^2	0.31	0.29	0.25	0.27
Clusters	76	76	76	76
Observations	513	513	512	513
Dependent variable: Gender gaps with correction for age profiles in preferences				
Birth decade	-0.012***	-0.017***	-0.019***	-0.011***
	(0.003)	(0.004)	(0.004)	(0.002)
R^2	0.29	0.31	0.28	0.29
Clusters	76	76	76	76
Observations	513	513	512	513
**Panel (b): World Values Survey**				
Dependent variable: Gender gaps without correction for age profiles in preferences				
Birth decade	-0.012***	0.003	-0.011***	-0.012***
	(0.002)	(0.004)	(0.002)	(0.002)
R^2	0.23	0.29	0.24	0.24
Clusters	106	77	106	106
Observations	1,792	720	1,791	1,780
Dependent variable: Gender gaps with correction for age profiles in preferences				
Birth decade	-0.012***	-0.009**	-0.008***	-0.010***
	(0.002)	(0.003)	(0.002)	(0.002)
R^2	0.27	0.25	0.26	0.27
Clusters	106	77	106	106
Observations	1,792	720	1,791	1,780

Note: This table shows coefficient estimates from ordinary least squares regressions of within-country gender preference gaps on linear birth decade trends. The coefficients reflect the estimated changes in gender preference gaps per birth decade (i.e., cohort trends). In contrast to the main results, the individual-level data were not weighted during age correction and data aggregation. Results are shown for 76 countries observed over 7 birth decades in the Global Preferences Survey (Panel a) and for 106 countries and territories observed over 7 birth decades and up to 7 survey waves during 1981–2022 in the World Values Survey (Panel b). An observation is a within-country gender gap per birth decade. The dependent variable is the absolute difference in means of preferences between men and women (top of Panels a and b) or the absolute difference in means of age-corrected preferences between men and women (bottom of Panels a and b), where means are separately calculated for each gender within country-, period-, birth decade-specific cells. Age-corrected preference measures have been purged of country-, gender-specific age profiles in the cross-sectional data from the Global Preferences Survey and of country-, period-, gender-specific age profiles in the longitudinal data from the World Values Survey. All preference measures are standardized to mean 0 and standard deviation 1 on the respective global samples such that gender preference gaps are expressed in standard deviations of the corresponding preference measures (see Methods). Specifications for the Global Preferences Survey include country fixed effects, whereas specifications for the World Values Survey include country-period fixed effects. For a list of countries included in the Global Preferences Survey and World Values Survey samples, see Tables S.1 and S.2. Standard errors are clustered at the country level and reported in parentheses. Asterisks indicate significance levels: * $p < 0.1$; ** $p < 0.05$; *** $p < 0.01$.

Table S.7: Estimated changes in gender gaps per birth decade: Results weighted by cell size

Gender gap in	Patience (1)	Risk-taking (2)	Trust (3)	Prosociality (4)
**Panel (a): Global Preferences Survey**				
Dependent variable: Gender gaps without correction for age profiles in preferences				
Birth decade	-0.015***	-0.015***	-0.008**	-0.011***
	(0.004)	(0.005)	(0.004)	(0.002)
R^2	0.37	0.32	0.28	0.24
Clusters	76	76	76	76
Observations	513	513	512	513
Dependent variable: Gender gaps with correction for age profiles in preferences				
Birth decade	-0.008**	-0.015***	-0.014***	-0.012***
	(0.003)	(0.003)	(0.003)	(0.002)
R^2	0.31	0.25	0.26	0.27
Clusters	76	76	76	76
Observations	513	513	512	513
**Panel (b): World Values Survey**				
Dependent variable: Gender gaps without correction for age profiles in preferences				
Birth decade	-0.006***	0.012***	-0.006***	-0.007***
	(0.002)	(0.004)	(0.002)	(0.002)
R^2	0.27	0.44	0.26	0.27
Clusters	106	77	106	106
Observations	1,792	720	1,791	1,780
Dependent variable: Gender gaps with correction for age profiles in preferences				
Birth decade	-0.008***	-0.006***	-0.006***	-0.007***
	(0.001)	(0.002)	(0.001)	(0.001)
R^2	0.32	0.22	0.31	0.32
Clusters	106	77	106	106
Observations	1,792	720	1,791	1,780

Note: This table shows coefficient estimates from ordinary least squares regressions of within-country gender preference gaps on linear birth decade trends. The coefficients reflect the estimated changes in gender preference gaps per birth decade (i.e., cohort trends) weighted by the number of individual-level observations per cell. Results are shown for 76 countries observed over 7 birth decades in the Global Preferences Survey (Panel a) and for 106 countries and territories observed over 7 birth decades and up to 7 survey waves during 1981–2022 in the World Values Survey (Panel b). An observation is a within-country gender gap per birth decade. The dependent variable is the absolute difference in means of preferences between men and women (top of Panels a and b) or the absolute difference in means of age-corrected preferences between men and women (bottom of Panels a and b), where means are separately calculated for each gender within country-, period-, birth decade-specific cells. Age-corrected preference measures have been purged of country-, gender-specific age profiles in the cross-sectional data from the Global Preferences Survey and of country-, period-, gender-specific age profiles in the longitudinal data from the World Values Survey. All preference measures are standardized to mean 0 and standard deviation 1 on the respective global samples such that gender preference gaps are expressed in standard deviations of the corresponding preference measures (see Methods). Specifications for the Global Preferences Survey include country fixed effects, whereas specifications for the World Values Survey include country-period fixed effects. For a list of countries included in the Global Preferences Survey and World Values Survey samples, see Tables S.1 and S.2. Standard errors are clustered at the country level and reported in parentheses. Asterisks indicate significance levels: * $p < 0.1$; ** $p < 0.05$; *** $p < 0.01$.

Table S.8: Estimated decline in gender gaps between 1950 and 1980 birth decades

	Patience	Risk-taking	Trust	Prosociality
	(1)	(2)	(3)	(4)
**Panel (a): Global Preferences Survey**				
Change in gender gaps (in %)	-23.1***	-21.6***	-35.2***	-32.0***
	(5.3)	(3.9)	(6.1)	(4.3)
**Panel (b): World Values Survey**				
Change in gender gaps (in %)	-18.8***	-8.6***	-11.3***	-21.6***
	(2.8)	(3.2)	(2.6)	(3.9)

Note: This table shows estimated changes in gender preference gaps in the Global Preferences Survey (Panel a) and the World Values Survey (Panel b) between the 1950 and 1980 birth decades. Values are calculated by combining the estimated cohort coefficients (derived from the regressions for age-corrected preference measures in Table **??**) with the uncorrected gender preference gaps in 1950 and then multiplying the results by 3. The estimated changes and their standard errors (in parentheses) are reported in percent.

Table S.9: Variation in gender gaps explained by cohort fixed effects and country fixed effects

Gender gap in	Patience		Risk-taking		Trust		Prosociality	
	Cohort FE	Country FE	Cohort FE	Country FE	Cohort FE	Country FE	Cohort FE	Country FE
	(1)	(2)	(3)	(4)	(5)	(6)	(7)	(8)
**Panel (a): Global Preferences Survey**								
Uncorrected preference measures								
R^2	0.073	0.249	0.041	0.202	0.050	0.183	0.092	0.141
Observations	513	513	513	513	512	512	513	513
Age-corrected preference measures								
R^2	0.094	0.258	0.107	0.194	0.124	0.171	0.145	0.150
Observations	513	513	513	513	512	512	513	513
**Panel (b): World Values Survey**								
Uncorrected preference measures								
R^2	0.077	0.070	0.067	0.158	0.062	0.098	0.078	0.085
Observations	1,792	1,792	720	720	1,791	1,791	1,780	1,780
Age-corrected preference measures								
R^2	0.069	0.074	0.058	0.161	0.043	0.104	0.088	0.082
Observations	1,792	1,792	720	720	1,791	1,791	1,780	1,780

Note: This table shows how much variation in within-country gender preference gaps is explained by cohort and country fixed effects (R^2), where cohorts correspond to birth decades. Results are shown for 76 countries observed over 7 birth decades in the Global Preferences Survey (Panel a) and for 106 countries and territories observed over 7 birth decades and up to 7 survey waves during 1981–2022 in the World Values Survey (Panel b). An observation is a within-country gender gap per birth decade. The dependent variable is the absolute difference in means of preferences between men and women (top of Panels a and b) or the absolute difference in means of age-corrected preferences between men and women (bottom of Panels a and b), where means are separately calculated for each gender within country-, period-, birth decade-specific cells. Age-corrected preference measures have been purged of country-, gender-specific age profiles in the Global Preferences Survey and of country-, period-, gender-specific age profiles in the World Values Survey, with preference measures in the World Values Survey additionally corrected for period fixed effects before aggregation.

Table S.10: Estimated changes in gender gaps per birth decade: Stable age profiles

Gender gap in	Patience<br>(1)	Risk-taking<br>(2)	Trust<br>(3)	Prosociality<br>(4)
Dependent variable: Gender gaps without correction for age profiles in preferences				
Birth decade	-0.012***	0.003	-0.011***	-0.012***
	(0.002)	(0.004)	(0.002)	(0.002)
R^2	0.23	0.29	0.24	0.24
Clusters	106	77	106	106
Observations	1,792	720	1,791	1,780
Dependent variable: Gender gaps with correction for age profiles in preferences				
Birth decade	-0.013***	-0.009***	-0.010***	-0.013***
	(0.002)	(0.003)	(0.002)	(0.002)
R^2	0.22	0.22	0.23	0.24
Clusters	106	77	106	106
Observations	1,792	720	1,791	1,780

Note: This table shows coefficient estimates from ordinary least squares regressions of within-country gender preference gaps on linear birth decade trends. The coefficients reflect the estimated changes in gender preference gaps per birth decade (i.e., cohort trends). Results are shown for 106 countries and territories observed over 7 birth decades and up to 7 survey waves during 1981–2022 in the World Values Survey. An observation is a within-country gender gap per birth decade. The dependent variable is the absolute difference in means of preferences (top panel) or the absolute difference in means of age-corrected preferences (bottom panel), where means are separately calculated for each gender within country-, period-, birth decade-specific cells. Age-corrected preference measures have been purged of country-, gender-specific age profiles. All preference measures are standardized to mean 0 and standard deviation 1 on the respective global samples such that gender preference gaps are expressed in standard deviations of the corresponding preference measures (see Methods). Specifications include country-period fixed effects. Standard errors are clustered at the country level and reported in parentheses. Asterisks indicate significance levels: * $p < 0.1$; ** $p < 0.05$; *** $p < 0.01$.

Table S.11: Estimated changes in gender gaps per birth decade: Cohort-specific age profiles

Gender gap in	Patience<br>(1)	Risk-taking<br>(2)	Trust<br>(3)	Prosociality<br>(4)
Dependent variable: Gender gaps without correction for age profiles in preferences				
Birth decade	-0.012***	0.003	-0.011***	-0.012***
	(0.002)	(0.004)	(0.002)	(0.002)
R^2	0.23	0.29	0.24	0.24
Clusters	106	77	106	106
Observations	1,792	720	1,791	1,780
Dependent variable: Gender gaps with correction for age profiles in preferences				
Birth decade	-0.008***	-0.005***	-0.007***	-0.010***
	(0.001)	(0.001)	(0.001)	(0.001)
R^2	0.31	0.45	0.34	0.31
Clusters	106	77	106	106
Observations	1,792	720	1,791	1,780

Note: This table shows coefficient estimates from ordinary least squares regressions of within-country gender preference gaps on linear birth decade trends. The coefficients reflect the estimated changes in gender preference gaps per birth decade (i.e., cohort trends). Results are shown for 106 countries and territories observed over 7 birth decades and up to 7 survey waves during 1981–2022 in the World Values Survey. An observation is a within-country gender gap per birth decade. The dependent variable is the absolute difference in means of preferences (top panel) or the absolute difference in means of age-corrected preferences (bottom panel), where means are separately calculated for each gender within country-, period-, birth decade-specific cells. Age-corrected preference measures have been purged of country-, cohort-, gender-specific age profiles. All preference measures are standardized to mean 0 and standard deviation 1 on the respective global samples such that gender preference gaps are expressed in standard deviations of the corresponding preference measures (see Methods). Specifications include country-period fixed effects. Standard errors are clustered at the country level and reported in parentheses. Asterisks indicate significance levels: * $p < 0.1$; ** $p < 0.05$; *** $p < 0.01$.

Table S.12: Estimated changes in gender gaps by age and cohort

Gender gap in	Patience (1)	Risk-taking (2)	Trust (3)	Prosociality (4)
**Panel (a): Age and cohort variation (no age correction)**				
Age group	0.013**	-0.009	-0.006	0.001
	(0.006)	(0.009)	(0.006)	(0.006)
Birth decade	-0.009	-0.010	-0.019***	-0.010*
	(0.006)	(0.009)	(0.006)	(0.005)
R^2	0.08	0.07	0.13	0.10
Clusters	77	68	77	77
Observations	11,520	4,561	11,323	11,435
**Panel (b): Age and cohort variation (age-corrected preference measures, stable age profiles)**				
Age group	0.011*	0.006	-0.006	-0.000
	(0.006)	(0.008)	(0.005)	(0.005)
Birth decade	-0.012*	-0.006	-0.021***	-0.013**
	(0.006)	(0.008)	(0.005)	(0.005)
R^2	0.08	0.06	0.13	0.09
Clusters	77	68	77	77
Observations	11,520	4,561	11,323	11,435
**Panel (c): Age and cohort variation (age-corrected preference measures, period-specific age profiles)**				
Age group	0.009	0.008	-0.004	0.002
	(0.006)	(0.008)	(0.005)	(0.005)
Birth decade	-0.013**	-0.005	-0.019***	-0.010*
	(0.005)	(0.008)	(0.005)	(0.005)
R^2	0.08	0.07	0.13	0.09
Clusters	77	68	77	77
Observations	11,520	4,561	11,323	11,435
**Panel (d): Age and cohort variation (age-corrected preference measures, period-specific age profiles)**				
Age group				
<25	-0.021	-0.013	0.068*	0.016
	(0.039)	(0.068)	(0.036)	(0.040)
25–34	-0.028	-0.017	0.051	0.002
	(0.033)	(0.055)	(0.032)	(0.031)
35–44	-0.031	-0.014	0.037	-0.006
	(0.025)	(0.043)	(0.028)	(0.026)
45–54	-0.012	-0.010	0.036*	-0.011
	(0.020)	(0.031)	(0.021)	(0.019)
55–64	-0.008	0.016	0.021	-0.006
	(0.013)	(0.024)	(0.016)	(0.015)
65+	ref.	ref.	ref.	ref.
Birth year	-0.017**	-0.007	-0.027***	-0.016**
(estimates × 10)	(0.008)	(0.013)	(0.007)	(0.008)
R^2	0.09	0.07	0.13	0.09
Clusters	77	68	77	77
Observations	11,520	4,561	11,323	11,435

Note: This table shows coefficient estimates from ordinary least squares regressions of within-country gender preference gaps on linear birth decade and age group trends (Panels a, b, and c) or on linear birth decade and arbitrary age group trends (Panel d). The coefficients reflect the estimated changes in gender preference gaps per birth decade (i.e., cohort trends) and per 10-year age group. Results are shown for 77 countries and territories observed over 7 birth decades (69 birth years) and up to 7 survey waves during 1981–2022 in the World Values Survey. An observation is a within-country gender gap per birth year. The dependent variable is the absolute difference in means of uncorrected preferences (Panel a) or the absolute difference in means of age-corrected preferences (Panels b, c, and d), where means are separately calculated for each gender within country-, period-, birth year-specific cells. Age-corrected preference measures have been either purged of country-, gender-specific age profiles (Panel b) or of country-, period-, gender-specific age profiles (Panels c and d). All preference measures are standardized to mean 0 and standard deviation 1 on the respective global samples such that gender preference gaps are expressed in standard deviations of the corresponding preference measures (see Methods). Specifications include country-period fixed effects. Standard errors are clustered at the country level and reported in parentheses. Asterisks indicate significance levels: * $p < 0.1$; ** $p < 0.05$; *** $p < 0.01$.

Table S.13: Gender gaps without age correction: Global Preferences Survey

Controlling for	Economic development (1)	Gender equality (2)	Cohort trends (3)	Full controls (4)	Cohort variation and fixed effects (5)
**Panel (a): Gender gap in patience**					
Log income per capita	0.023**	—	—	0.019**	—
	(0.010)	—	—	(0.009)	—
Gender equality	—	0.584***	—	0.459***	—
	—	(0.162)	—	(0.147)	—
Birth decade	—	—	-0.022***	-0.021***	-0.021***
	—	—	(0.005)	(0.005)	(0.005)
R^2	0.02	0.04	0.07	0.11	0.31
Clusters	75	70	70	70	70
Observations	507	475	475	475	475
**Panel (b): Gender gap in risk-taking**					
Log income per capita	0.011	—	—	0.016**	—
	(0.008)	—	—	(0.006)	—
Gender equality	—	0.158	—	0.057	—
	—	(0.208)	—	(0.202)	—
Birth decade	—	—	-0.015***	-0.015***	-0.015***
	—	—	(0.005)	(0.005)	(0.005)
R^2	0.00	0.00	0.03	0.04	0.23
Clusters	75	70	70	70	70
Observations	507	475	475	475	475
**Panel (c): Gender gap in trust**					
Log income per capita	-0.008	—	—	-0.012	—
	(0.008)	—	—	(0.010)	—
Gender equality	—	0.148	—	0.171	—
	—	(0.144)	—	(0.144)	—
Birth decade	—	—	-0.019***	-0.019***	-0.019***
	—	—	(0.005)	(0.005)	(0.005)
R^2	0.00	0.00	0.04	0.05	0.22
Clusters	75	70	70	70	70
Observations	506	474	474	474	474
**Panel (d): Gender gap in prosociality**					
Log income per capita	-0.009*	—	—	-0.010*	—
	(0.005)	—	—	(0.005)	—
Gender equality	—	-0.051	—	-0.022	—
	—	(0.093)	—	(0.085)	—
Birth decade	—	—	-0.013***	-0.014***	-0.014***
	—	—	(0.003)	(0.003)	(0.003)
R^2	0.01	0.00	0.06	0.07	0.20
Clusters	75	70	70	70	70
Observations	507	475	475	475	475

Note: This table shows coefficient estimates from ordinary least squares regressions of within-country gender preference gaps on economic development, gender equality, and linear birth decade trends. The cohort coefficients reflect the estimated changes in gender preference gaps per birth decade (i.e., cohort trends). Results are shown for 75 countries observed over 7 birth decades in the Global Preferences Survey. An observation is a within-country gender gap per birth decade. The dependent variable is the absolute difference in means of patience (Panel a), willingness to take risks (Panel b), trust (Panel c), or prosociality (Panel d), where means are separately calculated for each gender within country-, birth decade-specific cells. All preference measures are standardized to mean 0 and standard deviation 1 on the respective global samples such that gender preference gaps are expressed in standard deviations of the corresponding preference measures (see Methods). Economic development is measured with log 2012 real gross domestic product per capita in national accounts obtained from Penn World Tables (**?**). Gender equality is proxied by the World Economic Forum's gender parity index (**?**). Specification (5) includes country fixed effects. Standard errors are clustered at the country level and reported in parentheses. Asterisks indicate significance levels: * $p < 0.1$; ** $p < 0.05$; *** $p < 0.01$.

Table S.14: Gender gaps with age correction: Global Preferences Survey

Controlling for	Economic development (1)	Gender equality (2)	Cohort trends (3)	Full controls (4)	Cohort variation and fixed effects (5)
**Panel (a): Gender gap in patience**					
Log income per capita	0.014**	—	—	0.008	—
	(0.006)	—	—	(0.006)	—
Gender equality	—	0.257**	—	0.192*	—
	—	(0.109)	—	(0.106)	—
Birth decade	—	—	-0.015***	-0.015***	-0.015***
	—	—	(0.004)	(0.004)	(0.004)
R^2	0.02	0.01	0.06	0.08	0.32
Clusters	75	70	70	70	70
Observations	507	475	475	475	475
**Panel (b): Gender gap in risk-taking**					
Log income per capita	-0.017***	—	—	-0.018***	—
	(0.006)	—	—	(0.006)	—
Gender equality	—	-0.027	—	0.029	—
	—	(0.115)	—	(0.110)	—
Birth decade	—	—	-0.020***	-0.020***	-0.020***
	—	—	(0.004)	(0.004)	(0.004)
R^2	0.02	0.00	0.09	0.11	0.30
Clusters	75	70	70	70	70
Observations	507	475	475	475	475
**Panel (c): Gender gap in trust**					
Log income per capita	-0.010*	—	—	-0.010	—
	(0.006)	—	—	(0.007)	—
Gender equality	—	-0.074	—	-0.064	—
	—	(0.114)	—	(0.112)	—
Birth decade	—	—	-0.022***	-0.022***	-0.022***
	—	—	(0.004)	(0.004)	(0.004)
R^2	0.01	0.00	0.11	0.12	0.29
Clusters	75	70	70	70	70
Observations	506	474	474	474	474
**Panel (d): Gender gap in prosociality**					
Log income per capita	-0.009***	—	—	-0.012***	—
	(0.003)	—	—	(0.004)	—
Gender equality	—	-0.026	—	0.006	—
	—	(0.055)	—	(0.049)	—
Birth decade	—	—	-0.015***	-0.015***	-0.015***
	—	—	(0.002)	(0.002)	(0.002)
R^2	0.02	0.00	0.13	0.16	0.29
Clusters	75	70	70	70	70
Observations	507	475	475	475	475

Note: This table shows coefficient estimates from ordinary least squares regressions of within-country gender preference gaps on economic development, gender equality, and linear birth decade trends. The cohort coefficients reflect the estimated changes in gender preference gaps per birth decade (i.e., cohort trends). Results are shown for 75 countries observed over 7 birth decades in the Global Preferences Survey. An observation is a within-country gender gap per birth decade. The dependent variable is the absolute difference in means of patience (Panel a), willingness to take risks (Panel b), trust (Panel c), or prosociality (Panel d), where means are separately calculated for each gender within country-, birth decade-specific cells. Preference measures have been purged of country-, gender-specific age profiles. All preference measures are standardized to mean 0 and standard deviation 1 on the respective global samples such that gender preference gaps are expressed in standard deviations of the corresponding preference measures (see Methods). Economic development is measured with log 2012 real gross domestic product per capita in national accounts obtained from Penn World Tables (**?**). Gender equality is proxied by the World Economic Forum's gender parity index (**?**). Specification (5) includes country fixed effects. Standard errors are clustered at the country level and reported in parentheses. Asterisks indicate significance levels: * $p < 0.1$; ** $p < 0.05$; *** $p < 0.01$.

Table S.15: Gender gaps without age correction: World Values Survey

Controlling for	Economic development	Gender equality	Cohort trends	Full controls	Cohort variation and country–period fixed effects
	(1)	(2)	(3)	(4)	(5)
**Panel (a): Gender gap in patience**					
Log income per capita	-0.000	—	—	-0.002	—
	(0.004)	—	—	(0.005)	—
Gender equality	—	-0.122	—	-0.129	—
	—	(0.079)	—	(0.087)	—
Birth decade	—	—	-0.014***	-0.014***	-0.015***
	—	—	(0.003)	(0.003)	(0.003)
R^2	0.00	0.00	0.04	0.04	0.22
Clusters	103	85	85	85	85
Observations	1,751	1,037	1,037	1,037	1,037
**Panel (b): Gender gap in risk-taking**					
Log income per capita	0.008	—	—	0.012	—
	(0.010)	—	—	(0.013)	—
Gender equality	—	-0.147	—	-0.236	—
	—	(0.139)	—	(0.175)	—
Birth decade	—	—	0.003	0.003	0.003
	—	—	(0.005)	(0.005)	(0.005)
R^2	0.00	0.00	0.00	0.01	0.29
Clusters	75	69	69	69	69
Observations	707	655	655	655	655
**Panel (c): Gender gap in trust**					
Log income per capita	0.004	—	—	-0.005	—
	(0.004)	—	—	(0.006)	—
Gender equality	—	-0.061	—	-0.045	—
	—	(0.105)	—	(0.097)	—
Birth decade	—	—	-0.011***	-0.011***	-0.012***
	—	—	(0.003)	(0.003)	(0.003)
R^2	0.00	0.00	0.02	0.03	0.14
Clusters	103	85	85	85	85
Observations	1,750	1,031	1,031	1,031	1,031
**Panel (d): Gender gap in prosociality**					
Log income per capita	0.000	—	—	-0.006	—
	(0.004)	—	—	(0.005)	—
Gender equality	—	-0.104	—	-0.086	—
	—	(0.103)	—	(0.106)	—
Birth decade	—	—	-0.014***	-0.014***	-0.015***
	—	—	(0.003)	(0.003)	(0.003)
R^2	0.00	0.00	0.04	0.04	0.16
Clusters	103	85	85	85	85
Observations	1,739	1,037	1,037	1,037	1,037

Note: This table shows coefficient estimates from ordinary least squares regressions of within-country gender preference gaps on economic development, gender equality, and linear birth decade trends. The cohort coefficients reflect the estimated changes in gender preference gaps per birth decade (i.e., cohort trends). Results are shown for up to 103 countries and territories observed over up to 4 waves and up to 7 birth decades in the World Values Survey. An observation is a within-country gender gap per birth decade. The dependent variable is the absolute difference in means of patience (Panel a), willingness to take risks (Panel b), trust (Panel c), or prosociality (Panel d), where means are separately calculated for each gender within country-, period-, birth decade-specific cells. All preference measures are standardized to mean 0 and standard deviation 1 on the respective global samples such that gender preference gaps are expressed in standard deviations of the corresponding preference measures (see Methods). Economic development is measured with log 2012 real gross domestic product per capita in national accounts obtained from Penn World Tables (**?**). Gender equality is proxied by the World Economic Forum's gender parity index (**?**). Specification (5) includes country-period fixed effects. Standard errors are clustered at the country level and reported in parentheses. Asterisks indicate significance levels: * $p < 0.1$; ** $p < 0.05$; *** $p < 0.01$.

Table S.16: Gender gaps with age correction: World Values Survey

Controlling for	Economic development	Gender equality	Cohort trends	Full controls	Cohort variation and country–period fixed effects
	(1)	(2)	(3)	(4)	(5)
**Panel (a): Gender gap in patience**					
Log income per capita	-0.003	—	—	-0.004	—
	(0.002)	—	—	(0.004)	—
Gender equality	—	-0.132**	—	-0.123*	—
	—	(0.056)	—	(0.062)	—
Birth decade	—	—	-0.015***	-0.015***	-0.016***
	—	—	(0.002)	(0.002)	(0.002)
R^2	0.00	0.01	0.08	0.09	0.29
Clusters	103	85	85	85	85
Observations	1,751	1,037	1,037	1,037	1,037
**Panel (b): Gender gap in risk-taking**					
Log income per capita	-0.021***	—	—	-0.024**	—
	(0.007)	—	—	(0.009)	—
Gender equality	—	-0.105	—	0.070	—
	—	(0.077)	—	(0.104)	—
Birth decade	—	—	-0.007**	-0.007**	-0.008**
	—	—	(0.003)	(0.003)	(0.004)
R^2	0.03	0.00	0.01	0.05	0.25
Clusters	75	69	69	69	69
Observations	707	655	655	655	655
**Panel (c): Gender gap in trust**					
Log income per capita	0.002	—	—	0.001	—
	(0.003)	—	—	(0.005)	—
Gender equality	—	-0.059	—	-0.076	—
	—	(0.065)	—	(0.061)	—
Birth decade	—	—	-0.009***	-0.009***	-0.010***
	—	—	(0.002)	(0.002)	(0.002)
R^2	0.00	0.00	0.03	0.04	0.17
Clusters	103	85	85	85	85
Observations	1,750	1,031	1,031	1,031	1,031
**Panel (d): Gender gap in prosociality**					
Log income per capita	-0.001	—	—	-0.004	—
	(0.003)	—	—	(0.004)	—
Gender equality	—	0.014	—	0.022	—
	—	(0.061)	—	(0.067)	—
Birth decade	—	—	-0.012***	-0.012***	-0.013***
	—	—	(0.003)	(0.003)	(0.003)
R^2	0.00	0.00	0.05	0.05	0.14
Clusters	103	85	85	85	85
Observations	1,739	1,037	1,037	1,037	1,037

Note: This table shows coefficient estimates from ordinary least squares regressions of within-country gender preference gaps on economic development, gender equality, and linear birth decade trends. The cohort coefficients reflect the estimated changes in gender preference gaps per birth decade (i.e., cohort trends). Results are shown for up to 103 countries and territories observed over up to 4 waves and up to 7 birth decades in the World Values Survey. An observation is a within-country gender gap per birth decade. The dependent variable is the absolute difference in means of patience (Panel a), willingness to take risks (Panel b), trust (Panel c), or prosociality (Panel d), where means are separately calculated for each gender within country-, period-, birth decade-specific cells. Preference measures have been purged of country-, period-, gender-specific age profiles. All preference measures are standardized to mean 0 and standard deviation 1 on the respective global samples such that gender preference gaps are expressed in standard deviations of the corresponding preference measures (see Methods). Economic development is measured with log 2012 real gross domestic product per capita in national accounts obtained from Penn World Tables (**?**). Gender equality is proxied by the World Economic Forum's gender parity index (**?**). Specification (5) includes country-period fixed effects. Standard errors are clustered at the country level and reported in parentheses. Asterisks indicate significance levels: * $p < 0.1$; ** $p < 0.05$; *** $p < 0.01$.

Table S.17: Estimated changes in gender gaps of employment preferences per birth decade

Gender gap in	Employment preference for men over women when jobs are scarce	
	Without correction for age profiles<br>(1)	With correction for age profiles<br>(2)
Birth decade	0.011***<br>(0.003)	-0.013***<br>(0.002)
R^2	0.45	0.29
Clusters	105	105
Observations	1,756	1,756

Note: This table shows coefficient estimates from ordinary least squares regressions of within-country gender preference gaps on linear birth decade trends. The coefficients reflect the estimated changes in gender preference gaps per birth decade (i.e., cohort trends). Results are shown for 105 countries and territories observed over 7 birth decades and up to 7 survey waves during 1981–2022 in the World Values Survey. An observation is a within-country gender gap per birth decade. The dependent variable is the absolute difference in means of preferences between men and women (specification 1) or the absolute difference in means of age-corrected preferences between men and women (specification 2), where means are separately calculated for each gender within country-, period-, birth decade-specific cells. Age-corrected preference measures have been purged of country-, period-, gender-specific age profiles. The preference measure is standardized to mean 0 and standard deviation 1 on the global sample such that gender preference gaps are expressed in standard deviations (see Methods). All specifications include country-period fixed effects. For a list of countries included in the sample, see Table S.2. Standard errors are clustered at the country level and reported in parentheses. Asterisks indicate significance levels: * $p < 0.1$; ** $p < 0.05$; *** $p < 0.01$.

Table S.18: Estimated changes in gender gaps per birth decade: Accounting for gender attitudes

Gender gap in	Patience (1)	Risk-taking (2)	Trust (3)	Prosociality (4)
**Panel (a): Global Preferences Survey**				
Dependent variable: Gender gaps without correction for age profiles in preferences				
Birth decade	-0.021***	-0.018**	-0.024***	-0.010***
	(0.006)	(0.008)	(0.007)	(0.003)
Gap in gender attitudes	0.036	0.017	0.176*	0.104**
	(0.079)	(0.114)	(0.095)	(0.048)
R^2	0.28	0.26	0.26	0.21
Clusters	37	37	37	37
Observations	249	249	249	249
Dependent variable: Gender gaps with correction for age profiles in preferences				
Birth decade	-0.015***	-0.023***	-0.018***	-0.012***
	(0.005)	(0.004)	(0.004)	(0.003)
Gap in gender attitudes (age-corrected)	0.232**	0.336**	0.259**	-0.014
	(0.106)	(0.153)	(0.108)	(0.043)
R^2	0.31	0.40	0.36	0.29
Clusters	37	37	37	37
Observations	249	249	249	249
**Panel (b): World Values Survey**				
Dependent variable: Gender gaps without correction for age profiles in preferences				
Birth decade	-0.013***	0.002	-0.012***	-0.013***
	(0.002)	(0.004)	(0.002)	(0.002)
Gap in gender attitudes	0.079***	0.073	0.085***	0.119***
	(0.027)	(0.067)	(0.027)	(0.028)
R^2	0.23	0.29	0.25	0.26
Clusters	105	77	105	105
Observations	1,750	720	1,749	1,738
Dependent variable: Gender gaps with correction for age profiles in preferences				
Birth decade	-0.008***	-0.003	-0.005***	-0.006***
	(0.002)	(0.003)	(0.002)	(0.002)
Gap in gender attitudes (age-corrected)	0.306***	0.352***	0.175***	0.284***
	(0.038)	(0.077)	(0.033)	(0.062)
R^2	0.34	0.33	0.29	0.32
Clusters	105	77	105	105
Observations	1,750	720	1,749	1,738

Note: This table shows coefficient estimates from ordinary least squares regressions of within-country gender preference gaps on linear birth decade trends and gaps in gender attitudes. The coefficients reflect the estimated changes in gender preference gaps per birth decade (i.e., cohort trends). Gaps in gender attitudes are measured by gender gaps in respondents' agreement to the question whether men should have more right to work than women when jobs are scarce. Results are shown for 37 countries observed over 7 birth decades in the Global Preferences Survey (Panel a) and for 105 countries and territories observed over 7 birth decades and up to 7 survey waves during 1981–2022 in the World Values Survey (Panel b). An observation is a within-country gender gap per birth decade. The dependent variable is the absolute difference in means of preferences between men and women (top of Panels a and b) or the absolute difference in means of age-corrected preferences between men and women (bottom of Panels a and b), where means are separately calculated for each gender within country-, period-, birth decade-specific cells. Age-corrected preference measures and gaps in gender attitudes have been purged of country-, gender-specific age profiles in the cross-sectional data from the Global Preferences Survey and of country-, period-, gender-specific age profiles in the longitudinal data from the World Values Survey. All preference measures are standardized to mean 0 and standard deviation 1 on the respective global samples such that gender preference gaps are expressed in standard deviations of the corresponding preference measures (see Methods). Specifications for the Global Preferences Survey include country fixed effects, whereas specifications for the World Values Survey include country-period fixed effects. For a list of countries included in the Global Preferences Survey and World Values Survey samples, see Tables S.1 and S.2. Standard errors are clustered at the country level and reported in parentheses. Asterisks indicate significance levels: * $p < 0.1$; ** $p < 0.05$; *** $p < 0.01$.

Table S.19: Estimated changes in gender gaps per birth decade: Accounting for historical determinants of preference formation in the Global Preferences Survey

Controlling for	Patience		Risk-taking		Trust		Prosociality	
	(1)	(2)	(3)	(4)	(5)	(6)	(7)	(8)
**Panel (a): Dependent variable: Gender gaps without correction for age profiles in preferences**								
Birth decade	-0.021***	-0.020***	-0.017***	-0.017***	-0.017***	-0.018***	-0.014***	-0.014***
	(0.004)	(0.005)	(0.005)	(0.005)	(0.005)	(0.005)	(0.003)	(0.003)
Time since Neolithic Revolution	—	-0.010	—	-0.020*	—	-0.018*	—	-0.006
	—	(0.011)	—	(0.011)	—	(0.009)	—	(0.005)
Plow	—	0.008	—	0.006	—	-0.014	—	0.001
	—	(0.015)	—	(0.013)	—	(0.015)	—	(0.009)
Absolute latitude	—	0.014	—	0.020	—	0.014	—	0.002
	—	(0.019)	—	(0.014)	—	(0.016)	—	(0.008)
Mean elevation	—	-0.004	—	0.007	—	0.019	—	0.003
	—	(0.015)	—	(0.013)	—	(0.012)	—	(0.008)
Terrain roughness	—	-0.006	—	0.001	—	-0.009	—	0.008
	—	(0.014)	—	(0.011)	—	(0.010)	—	(0.005)
Landlocked	—	0.009	—	0.022**	—	-0.004	—	0.002
	—	(0.014)	—	(0.010)	—	(0.009)	—	(0.007)
Soil suitability	—	-0.003	—	0.008	—	-0.001	—	-0.005
	—	(0.018)	—	(0.014)	—	(0.016)	—	(0.009)
Crop yield	—	0.001	—	-0.029	—	-0.001	—	0.005
	—	(0.018)	—	(0.019)	—	(0.015)	—	(0.008)
Crop cycle	—	-0.011	—	0.009	—	-0.024	—	-0.006
	—	(0.021)	—	(0.018)	—	(0.019)	—	(0.013)
R^2	0.06	0.09	0.03	0.08	0.04	0.08	0.06	0.07
Clusters	75	75	75	75	75	75	75	75
Observations	507	507	507	507	506	506	507	507
**Panel (b): Dependent variable: Gender gaps with correction for age profiles in preferences**								
Birth decade	-0.015***	-0.014***	-0.021***	-0.022***	-0.022***	-0.022***	-0.014***	-0.015***
	(0.003)	(0.003)	(0.004)	(0.004)	(0.004)	(0.004)	(0.002)	(0.002)
Time since Neolithic Revolution	—	0.001	—	0.005	—	-0.001	—	-0.007*
	—	(0.008)	—	(0.008)	—	(0.009)	—	(0.004)
Plow	—	-0.008	—	-0.027**	—	-0.013	—	0.005
	—	(0.011)	—	(0.013)	—	(0.014)	—	(0.005)
Absolute latitude	—	0.008	—	0.003	—	-0.008	—	-0.010*
	—	(0.015)	—	(0.011)	—	(0.011)	—	(0.006)
Mean elevation	—	-0.009	—	-0.008	—	-0.009	—	0.003
	—	(0.007)	—	(0.010)	—	(0.009)	—	(0.006)
Terrain roughness	—	-0.012	—	-0.002	—	-0.004	—	-0.003
	—	(0.008)	—	(0.008)	—	(0.008)	—	(0.005)
Landlocked	—	-0.011*	—	0.010	—	0.011	—	0.004
	—	(0.006)	—	(0.009)	—	(0.007)	—	(0.005)
Soil suitability	—	0.003	—	0.008	—	0.003	—	0.007
	—	(0.013)	—	(0.013)	—	(0.014)	—	(0.006)
Crop yield	—	-0.006	—	-0.009	—	-0.017	—	0.001
	—	(0.010)	—	(0.014)	—	(0.014)	—	(0.006)
Crop cycle	—	0.005	—	0.003	—	0.002	—	-0.007
	—	(0.012)	—	(0.016)	—	(0.010)	—	(0.007)
R^2	0.06	0.10	0.10	0.13	0.11	0.14	0.13	0.15
Clusters	75	75	75	75	75	75	75	75
Observations	507	507	507	507	506	506	507	507

Note: This table shows coefficient estimates from ordinary least squares regressions of within-country gender preference gaps on linear birth decade trends and controls for historical determinants of preference formation. The cohort coefficients reflect the estimated changes in gender preference gaps per birth decade (i.e., cohort trends). Results are shown for 75 countries observed over 7 birth decades in the Global Preferences Survey. An observation is a within-country gender gap per birth decade. The dependent variable is the absolute difference in means of preferences between men and women (Panel a) or the absolute difference in means of age-corrected preferences between men and women (Panel b), where means are separately calculated for each gender within country-, birth decade-specific cells. Age-corrected preference measures have been purged of country-, gender-specific age profiles. All preference measures are standardized to mean 0 and standard deviation 1 on the respective global samples such that gender preference gaps are expressed in standard deviations of the corresponding preference measures (see Methods). For a list of countries included in the sample, see Table S.1. Historical control variables are obtained from **?** and normalized to mean 0 and standard deviation 1. Standard errors are clustered at the country level and reported in parentheses. Asterisks indicate significance levels: * $p < 0.1$; ** $p < 0.05$; *** $p < 0.01$.

Table S.20: Estimated changes in gender gaps per birth decade: Accounting for historical determinants of preference formation in the World Values Survey

Controlling for	Patience		Risk-taking		Trust		Prosociality	
	(1)	(2)	(3)	(4)	(5)	(6)	(7)	(8)
**Panel (a): Dependent variable: Gender gaps without correction for age profiles in preferences**								
Birth decade	-0.010***	-0.010***	0.003	0.003	-0.009***	-0.009***	-0.010***	-0.010***
	(0.002)	(0.002)	(0.004)	(0.004)	(0.002)	(0.002)	(0.002)	(0.002)
Time since Neolithic Revolution	—	-0.001	—	-0.001	—	-0.008*	—	0.003
	—	(0.005)	—	(0.013)	—	(0.005)	—	(0.005)
Plow	—	0.012*	—	0.008	—	0.012	—	-0.006
	—	(0.006)	—	(0.020)	—	(0.008)	—	(0.008)
Absolute latitude	—	-0.004	—	0.018	—	0.003	—	-0.008
	—	(0.006)	—	(0.014)	—	(0.007)	—	(0.008)
Mean elevation	—	-0.007	—	-0.012	—	0.006	—	-0.007
	—	(0.006)	—	(0.015)	—	(0.005)	—	(0.005)
Terrain roughness	—	0.009	—	0.019	—	-0.005	—	0.005
	—	(0.007)	—	(0.015)	—	(0.005)	—	(0.005)
Landlocked	—	0.009**	—	0.031**	—	0.009*	—	0.011*
	—	(0.004)	—	(0.013)	—	(0.005)	—	(0.006)
Soil suitability	—	-0.006	—	-0.014	—	-0.005	—	-0.004
	—	(0.007)	—	(0.011)	—	(0.006)	—	(0.007)
Crop yield	—	0.006	—	0.012	—	0.000	—	-0.001
	—	(0.006)	—	(0.013)	—	(0.006)	—	(0.008)
Crop cycle	—	-0.003	—	-0.007	—	-0.005	—	-0.010
	—	(0.004)	—	(0.010)	—	(0.009)	—	(0.012)
R^2	0.02	0.03	0.00	0.03	0.02	0.03	0.02	0.04
Clusters	98	98	72	72	98	98	98	98
Observations	1681	1681	675	675	1680	1680	1669	1669
**Panel (b): Dependent variable: Gender gaps with correction for age profiles in preferences**								
Birth decade	-0.009***	-0.010***	-0.008**	-0.008**	-0.006***	-0.006***	-0.008***	-0.008***
	(0.002)	(0.002)	(0.003)	(0.003)	(0.002)	(0.002)	(0.002)	(0.002)
Time since Neolithic Revolution	—	0.001	—	-0.011*	—	-0.003	—	0.004
	—	(0.003)	—	(0.007)	—	(0.003)	—	(0.003)
Plow	—	0.008**	—	0.009	—	0.005	—	-0.005
	—	(0.004)	—	(0.010)	—	(0.006)	—	(0.004)
Absolute latitude	—	-0.010**	—	-0.013*	—	0.004	—	-0.002
	—	(0.004)	—	(0.007)	—	(0.005)	—	(0.004)
Mean elevation	—	-0.002	—	0.003	—	0.004	—	-0.009***
	—	(0.004)	—	(0.007)	—	(0.005)	—	(0.003)
Terrain roughness	—	0.001	—	-0.007	—	-0.007*	—	0.007**
	—	(0.004)	—	(0.007)	—	(0.004)	—	(0.003)
Landlocked	—	0.007**	—	0.022**	—	0.006	—	0.011***
	—	(0.003)	—	(0.008)	—	(0.004)	—	(0.004)
Soil suitability	—	0.005	—	-0.002	—	0.001	—	-0.006
	—	(0.004)	—	(0.004)	—	(0.004)	—	(0.004)
Crop yield	—	-0.001	—	0.003	—	-0.006	—	-0.002
	—	(0.005)	—	(0.006)	—	(0.005)	—	(0.004)
Crop cycle	—	0.000	—	-0.013**	—	-0.001	—	0.007
	—	(0.004)	—	(0.006)	—	(0.006)	—	(0.004)
R^2	0.04	0.05	0.02	0.07	0.01	0.03	0.02	0.04
Clusters	98	98	72	72	98	98	98	98
Observations	1681	1681	675	675	1680	1680	1669	1669

Note: This table shows coefficient estimates from ordinary least squares regressions of within-country gender preference gaps on linear birth decade trends and controls for historical determinants of preference formation. The cohort coefficients reflect the estimated changes in gender preference gaps per birth decade (i.e., cohort trends). Results are shown for 98 countries over 7 birth decades and up to 7 survey waves during 1981–2022 in the World Values Survey. An observation is a within-country gender gap per birth decade. The dependent variable is the absolute difference in means of preferences between men and women (Panel a) or the absolute difference in means of age-corrected preferences between men and women (Panel b), where means are separately calculated for each gender within country-, period-, birth decade-specific cells. Age-corrected preference measures have been purged of country-, period-, gender-specific age profiles. All preference measures are standardized to mean 0 and standard deviation 1 on the respective global samples such that gender preference gaps are expressed in standard deviations of the corresponding preference measures (see Methods). For a list of countries included in the sample, see Table S.2. Historical control variables are obtained from **?** and normalized to mean 0 and standard deviation 1. Standard errors are clustered at the country level and reported in parentheses. Asterisks indicate significance levels: * $p < 0.1$; ** $p < 0.05$; *** $p < 0.01$.

Table S.21: Estimated changes in gender gaps per birth decade: Birth-decade trends interacted with select determinants of gender gaps in the Global Preferences Survey

Controlling for	Patience		Risk-taking		Trust		Prosociality	
	(1)	(2)	(3)	(4)	(5)	(6)	(7)	(8)
**Panel (a): Dependent variable: Gender gaps without correction for age profiles in preferences**								
Birth decade	-0.020***	-0.020***	-0.017***	-0.018***	-0.017***	-0.016***	-0.014***	-0.013***
	(0.004)	(0.005)	(0.005)	(0.005)	(0.004)	(0.004)	(0.003)	(0.002)
Birth decade × plow	0.007	—	0.013***	—	0.016***	—	0.009**	—
	(0.004)	—	(0.005)	—	(0.004)	—	(0.004)	—
Birth decade × absolute latitude	—	0.003	—	-0.001	—	0.016***	—	0.008***
	—	(0.005)	—	(0.005)	—	(0.004)	—	(0.003)
Marginal effect at 25th percentile	-0.024***	-0.024***	-0.026***	-0.017**	-0.027***	-0.034***	-0.020***	-0.022***
	(0.005)	(0.006)	(0.006)	(0.007)	(0.005)	(0.006)	(0.004)	(0.005)
Marginal effect at 75th percentile	-0.016***	-0.018***	-0.009	-0.018***	-0.007	-0.006	-0.008***	-0.008***
	(0.006)	(0.007)	(0.005)	(0.006)	(0.005)	(0.006)	(0.003)	(0.003)
R^2	0.31	0.31	0.25	0.24	0.25	0.25	0.23	0.23
Clusters	75	76	75	76	75	76	75	76
Observations	507	513	507	513	506	512	507	513
**Panel (b): Dependent variable: Gender gaps with correction for age profiles in preferences**								
Birth decade	-0.014***	-0.013***	-0.021***	-0.020***	-0.023***	-0.022***	-0.015***	-0.014***
	(0.003)	(0.003)	(0.004)	(0.004)	(0.004)	(0.004)	(0.002)	(0.002)
Plow	0.008***	—	0.011***	—	0.009**	—	0.005**	—
	(0.003)	—	(0.004)	—	(0.003)	—	(0.002)	—
Absolute latitude	—	0.009**	—	0.008**	—	0.010***	—	0.007***
	—	(0.004)	—	(0.004)	—	(0.003)	—	(0.002)
Marginal effect at 25th percentile	-0.019***	-0.024***	-0.029***	-0.029***	-0.028***	-0.033***	-0.018***	-0.022***
	(0.004)	(0.004)	(0.005)	(0.006)	(0.004)	(0.006)	(0.002)	(0.003)
Marginal effect at 75th percentile	-0.008**	-0.008	-0.014***	-0.015***	-0.017***	-0.015***	-0.012***	-0.010***
	(0.004)	(0.005)	(0.004)	(0.004)	(0.005)	(0.004)	(0.002)	(0.002)
R^2	0.32	0.34	0.32	0.31	0.31	0.31	0.30	0.32
Clusters	75	76	75	76	75	76	75	76
Observations	507	513	507	513	506	512	507	513

Note: This table shows coefficient estimates from ordinary least squares regressions of within-country gender preference gaps on linear birth decade trends interacted with historical plow use or absolute latitude. The cohort coefficients reflect the estimated changes in gender preference gaps per birth decade (i.e., cohort trends). Results are shown for 75 countries observed over 7 birth decades in the Global Preferences Survey. An observation is a within-country gender gap per birth decade. The dependent variable is the absolute difference in means of preferences between men and women (Panel a) or the absolute difference in means of age-corrected preferences between men and women (Panel b), where means are separately calculated for each gender within country-, birth decade-specific cells. Age-corrected preference measures have been purged of country-, gender-specific age profiles. All preference measures are standardized to mean 0 and standard deviation 1 on the respective global samples such that gender preference gaps are expressed in standard deviations of the corresponding preference measures (see Methods). For a list of countries included in the sample, see Table S.1. Historical control variables are obtained from **?** and normalized to mean 0 and standard deviation 1. All specifications include country fixed effects. Marginal effects for birth decade trends are reported for plow use and absolute latitude at the 25th and 75th percentiles. Standard errors are clustered at the country level and reported in parentheses. Asterisks indicate significance levels: * $p < 0.1$; ** $p < 0.05$; *** $p < 0.01$.

Table S.22: Estimated changes in gender gaps per birth decade: Birth-decade trends interacted with select determinants of gender gaps in the World Values Survey

Controlling for	Patience		Risk-taking		Trust		Prosociality	
	(1)	(2)	(3)	(4)	(5)	(6)	(7)	(8)
**Panel (a): Dependent variable: Gender gaps without correction for age profiles in preferences**								
Birth decade	-0.012***	-0.011***	0.002	0.003	-0.010***	-0.008***	-0.010***	-0.010***
	(0.002)	(0.002)	(0.004)	(0.004)	(0.002)	(0.002)	(0.002)	(0.002)
Birth decade × plow	0.002	—	0.014***	—	0.008***	—	0.005***	—
	(0.002)	—	(0.005)	—	(0.002)	—	(0.002)	—
Birth decade × absolute latitude	—	0.008***	—	0.007	—	0.006***	—	0.006***
	—	(0.002)	—	(0.005)	—	(0.002)	—	(0.002)
Marginal effect at 25th percentile	-0.013***	-0.018***	-0.005	-0.004	-0.014***	-0.015***	-0.013***	-0.016***
	(0.003)	(0.003)	(0.005)	(0.007)	(0.002)	(0.003)	(0.002)	(0.002)
Marginal effect at 75th percentile	-0.010***	-0.006***	0.011**	0.007*	-0.005*	-0.005**	-0.007***	-0.006***
	(0.003)	(0.002)	(0.005)	(0.004)	(0.003)	(0.002)	(0.002)	(0.002)
R^2	0.10	0.11	0.27	0.25	0.12	0.12	0.14	0.14
Clusters	101	105	74	76	101	105	101	105
Observations	1,741	1,786	701	714	1,740	1,785	1,729	1,774
**Panel (b): Dependent variable: Gender gaps with correction for age profiles in preferences**								
Birth decade	-0.010***	-0.010***	-0.009***	-0.007**	-0.007***	-0.006***	-0.008***	-0.008***
	(0.002)	(0.002)	(0.003)	(0.003)	(0.002)	(0.002)	(0.002)	(0.002)
Plow	0.004**	—	0.012***	—	0.005***	—	0.004**	—
	(0.002)	—	(0.004)	—	(0.002)	—	(0.002)	—
Absolute latitude	—	0.007***	—	0.012***	—	0.006***	—	0.004***
	—	(0.001)	—	(0.003)	—	(0.002)	—	(0.001)
Marginal effect at 25th percentile	-0.013***	-0.017***	-0.015***	-0.019***	-0.009***	-0.011***	-0.010***	-0.012***
	(0.002)	(0.002)	(0.004)	(0.004)	(0.001)	(0.002)	(0.002)	(0.002)
Marginal effect at 75th percentile	-0.008***	-0.006***	-0.001	-0.000	-0.004*	-0.003	-0.006***	-0.006***
	(0.002)	(0.002)	(0.003)	(0.003)	(0.002)	(0.002)	(0.002)	(0.002)
R^2	0.13	0.14	0.24	0.24	0.13	0.13	0.12	0.12
Clusters	101	105	74	76	101	105	101	105
Observations	1,741	1,786	701	714	1,740	1,785	1,729	1,774

Note: This table shows coefficient estimates from ordinary least squares regressions of within-country gender preference gaps on linear birth decade trends interacted with historical plow use or absolute latitude. The cohort coefficients reflect the estimated changes in gender preference gaps per birth decade (i.e., cohort trends). Results are shown for 106 countries and territories observed over 7 birth decades and up to 7 survey waves during 1981–2022 in the World Values Survey. An observation is a within-country gender gap per birth decade. The dependent variable is the absolute difference in means of preferences between men and women (Panel a) or the absolute difference in means of age-corrected preferences between men and women (Panel b), where means are separately calculated for each gender within country-, period-, birth decade-specific cells. Age-corrected preference measures have been purged of country-, period-, gender-specific age profiles. All preference measures are standardized to mean 0 and standard deviation 1 on the respective global samples such that gender preference gaps are expressed in standard deviations of the corresponding preference measures (see Methods). For a list of countries included in the sample, see Table S.2. Historical control variables are obtained from **?** and normalized to mean 0 and standard deviation 1. All specifications include country-period fixed effects. Marginal effects for birth decade trends are reported for plow use and absolute latitude at the 25th and 75th percentiles. Standard errors are clustered at the country level and reported in parentheses. Asterisks indicate significance levels: * $p < 0.1$; ** $p < 0.05$; *** $p < 0.01$.
